# Productivity loss associated with type 2 diabetes in China: the trend and projection from 1990 to 2050

**DOI:** 10.7189/jogh.16.04310

**Published:** 2026-09-25

**Authors:** Yiwen Zhang, Nianwei Wu, Haojie Zhang, Peige Song, Jing Li, Sheyu Li

**Affiliations:** 1 Department of Endocrinology and Metabolism, West China Hospital, Sichuan University, Chengdu, China; 2 School of Public Health, Zhejiang University School of Medicine, Hangzhou, China

**Keywords:** diabetes, productivity adjusted life years, projection, disease burden, economic burden

## Abstract

**Background:**

Type 2 diabetes (T2D) has become increasingly prevalent among young adults in their prime working years. This study aimed to estimate the productivity-adjusted life years (PALYs) loss associated with T2D among working-age Chinese adults from 1990 to 2021, with projections to 2050.

**Methods:**

Leveraging data from the Global Burden of Disease (GBD) 2021 database, the United Nations, and the China Chronic Disease and Risk Factors Surveillance study, we quantified individual- and population-level PALYs loss associated with T2D between 1990 and 2021 and projected trends through 2050 using a projection model. Loss of PALYs was calculated by combining productivity losses from excess mortality (years of life lost, YLL) with reductions in labour force participation, presenteeism, and absenteeism (years of productivity lost, YPL). Gross domestic product (GDP) loss was estimated by valuing each lost PALY using China’s annual GDP per worker.

**Results:**

In 2021, 39.8 million working-age Chinese men and 23.3 million women had T2D. Over their working lifetime, T2D was associated with 53.42 (95% uncertainty interval (UI) = 51.25–55.59) million PALYs loss in men and 33.56 (95% UI = 31.59–35.52) million in women, equivalent to 1.34 (95% UI = 1.29–1.40) and 1.44 (95% UI = 1.36–1.53) PALYs loss per man and woman, respectively. Loss of GDP reached USD 1035.31 billion for men and USD 650.38 billion for women. Total PALYs loss is projected to increase from 1990 to 2050, although per-person losses decline in both sexes. Women with T2D experience greater per-person PALYs loss than men in 2030 and 2050.

**Conclusions:**

T2D among working-age population is associated with substantial PALY and economic losses, underscoring urgent need for targeted interventions.

Approximately 277.1 million men and 259.4 million women were living with diabetes worldwide in 2021 [1]. Among them, more than 60% were of working age, contributing economic value to their countries, families and themselves [1]. Diabetes complications, including microvascular and macrovascular conditions [2–4], not only impair quality of life but also reduce work productivity through absenteeism (absence from work due to illness), presenteeism (reduced efficiency while at work), and early dropout from the workforce. China, which has the largest diabetes population and labour force globally [5,6], faces a particularly substantial public health and economic challenge. Population aging is gradually shrinking the labour force, while diabetes prevalence among adults is projected to rise through 2030 [5,7,8]. One study estimated the productivity adjusted life years (PALYs) lost due to diabetes in China under 2017 conditions; however, it did not assess longitudinal trends or provide future projections [9].

To address population aging, China has implemented a national strategy to delay the statutory retirement age [10]. Extending working life may increase the potential productive contribution of older adults, but it may also increase the number of working years exposed to diabetes-related productivity loss if healthy working life expectancy does not improve in parallel. It remains unclear how individual- and population-level productivity losses among working-age diabetes population in China have evolved over the past three decades, and how these losses are likely to change through 2050 under current demographic and policy trends. Timely updating and forecasting the diabetes-related productivity losses, accounting for demographic and economic changes, is pivotal for evidence-based policy making, resource allocation, and achieving the health-related targets of United Nations Sustainable Development Goal 3 [11]. This study thus aimed to estimate the individual- and population-level PALYs loss associated with type 2 diabetes among working-age adults in China from 1990 to 2021 and to project their trends through 2050.

## METHODS

Leveraging data from the Global Burden of Disease (GBD) 2021 database, the United Nations, and the China Chronic Disease and Risk Factors Surveillance study, we estimated individual- and population-level lifetime productivity losses among people prevalent with type 2 diabetes in each year from 1990 to 2021, and projected trends through 2050. To complement the remaining-lifetime estimates, we additionally quantified annual cross-sectional population-level PALYs and associated GDP losses for each calendar year from 1990 to 2050. Productivity losses consisted of:

• losses due to premature mortality associated with type 2 diabetes (years of life lost, YLL); and

• losses due to morbidity (years of productivity lost, YPL), including absenteeism, presenteeism, and reduced labour force participation (LFP).

Loss of PALYs was defined as the sum of YLL and YPL. The statutory retirement age in China is 60 years for males, 55 years for female professionals (*e.g.* teachers, medical personnel and other professionals), and 50 years for other female workers [12]. However, due to data availability, retirement age was conservatively assumed to be 59 years for males and 54 years for females.

We estimated the age- and sex-specific prevalence and the number of individuals with type 2 diabetes from 2021 to 2050 using an illness-death model. The model ran until the year 2089 to allow estimation of YLL, YPL and PALYs lost until age 59 years for a person who was 20 years old in 2050. The flowchart outlines the modelling framework used to estimate productivity-adjusted life years lost associated with type 2 diabetes (Figure S1 in the **Online Supplementary Document**).

### Data sources

Age group-, sex-, and year-specific incidence and prevalence of type 2 diabetes, as well as all-cause mortality for the general population from 1990 to 2021, were obtained from the GBD 2021 study. Prevalence estimates for 2021–2050 were projected based on 2021 data (Table S1 in the **Online Supplementary Document**). Population estimates and projections were sourced from the United Nations (UN) Department of Economic and Social Affairs/Population Division [13]. Future prospects were based on a demographic framework that incorporates fertility, mortality, and international migration [14]. Consistent with previous research, we applied the medium-variant population assumption, which assumes continued declines in fertility and age-specific mortality [15]. Key model inputs were derived from these data sources (Table S2 in the **Online Supplementary Document**).

This study adhered to the Guidelines for Accurate and Transparent Health Estimates Reporting (GATHER) (Checklist S1 in the **Online Supplementary Document**) [16]. The study didn’t involve any personal information. Consequently, no ethical approval was required for the study.

### Projection of type 2 diabetes prevalence

We applied an illness-death model to project the prevalence of type 2 diabetes in China from 2021 to 2050, dividing the population into three states: ‘healthy’, ‘diabetes’, and ‘dead’ [17]. Transitions between these states were characterised by the incidence rate (healthy → diabetes) and the mortality rates (healthy → death; diabetes → death) [17] (Figure S2 in the **Online Supplementary Document**). Due to data availability, we used the general population mortality rate *m* and the mortality rate ratio (MRR) comparing individuals with *vs.* without type 2 diabetes as inputs for the model, rather than the absolute mortality rates for each group. This yields the partial differential equation:



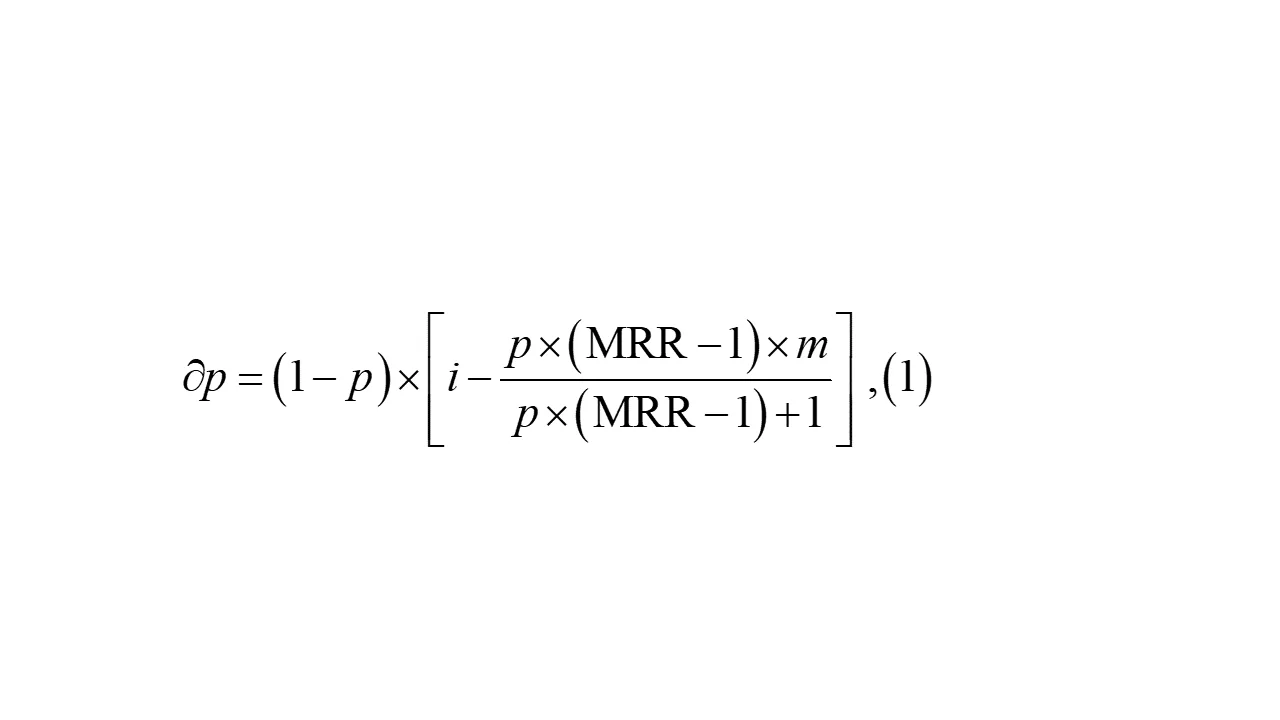



where *p* represents the prevalence of type 2 diabetes, *i* is the incidence, and ∂*p* represents the temporal change in prevalence. All parameters were modelled as time-dependent to account for assumed future trends in incidence, general mortality and MRR.

To estimate the future age- and sex-specific prevalence, the partial differential equation was solved by integration using nationally representative input data. We used the age- and sex-specific prevalence and incidence of type 2 diabetes as well as the general population mortality in 2021 in the GBD database as the initial parameters. Estimates of MRR were derived directly from the China Chronic Disease and Risk Factors Surveillance, a nationally representative study that assessed baseline glycaemic status at enrolment and prospectively ascertained mortality through 2019 [18]. Evidence indicates that mortality among individuals with type 2 diabetes decreases faster than in those without, resulting in a declining MRR [19,20]. Accordingly, we assumed annual MRR reductions of 0.60% for women and 0.72% for men from 2021 to 2089, consistent with previous studies [21].

### Calculation of productivity losses on the individual and population level

Years of life lost (YLL) were defined as the difference in remaining life expectancy up to retirement age between individuals with and without type 2 diabetes. The measure of YLL is defined by the following equation:



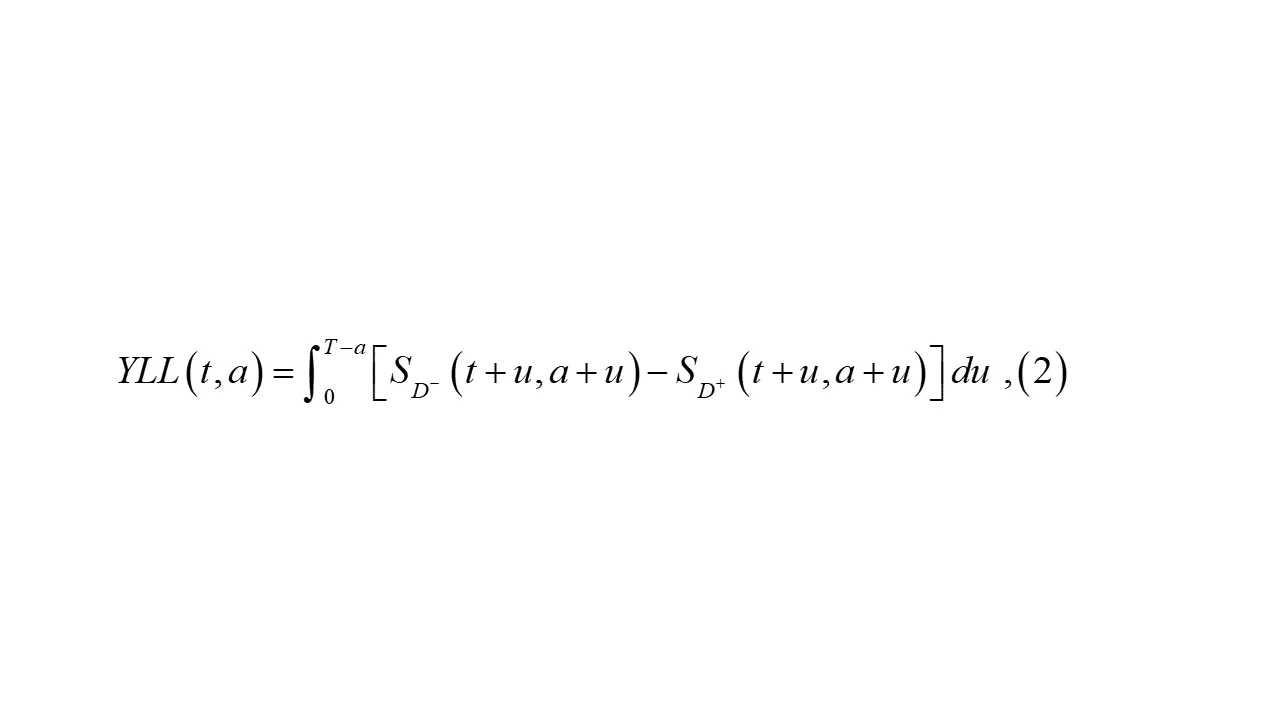



where *S_D_-* and *S_D+_* are the survival functions of people without and with type 2 diabetes, *t* is the calendar time, *a* is age, *T* is the statutory retirement age and *du* is the differential of the integration variable *u*. Productivity losses were accumulated only over working ages, with the integration domain restricted to *a*≤*T*, reflecting that individuals are assumed to exit the labour force upon reaching retirement age. This measure of YLL reflects the productivity loss due to early death in individuals with type 2 diabetes who would otherwise have remained in the labour force.

YPL comprised three components: absenteeism, presenteeism, and labour force dropout:

*YPL = absenteeism + presenteeism + labour force dropout (3)*

Labour force dropout was measured as the percentage shortfall in labour force participation among individuals with type 2 diabetes relative to those without type 2 diabetes. This ranged from 7.0% in women and 5.2% in men with diabetes aged 20–39 years to 12.8% in women and 8.3% in men with diabetes more than 40 years, respectively [22]. Absenteeism was defined as the excess number of work-loss days per year among individuals with type 2 diabetes relative to those without type 2 diabetes and was expressed as a percentage of total annual working days. Based on sex-specific estimates, the annual excess absenteeism was set at 2.1 workdays for women and 0.9 workdays for men, corresponding to productivity reductions of 0.9% and 0.4%, respectively, using China’s maximum of 245 working days per year [23]. Presenteeism was defined as reduced productivity while working and expressed as a percentage of total productivity. The diabetes-associated presenteeism was assumed to be 1.0% and 0.6% in women and men, respectively [22]. We combined the percentage of workdays lost (absenteeism) and the reduced productivity at work (presenteeism) into a single productivity loss weight (PLW). YPL for an individual with type 2 diabetes were then estimated by weighting each year until retirement by the corresponding shortfall in labour force participation (ΔLFP), absenteeism, and presenteeism, while accounting for survival probability. YPL were calculated as follows:



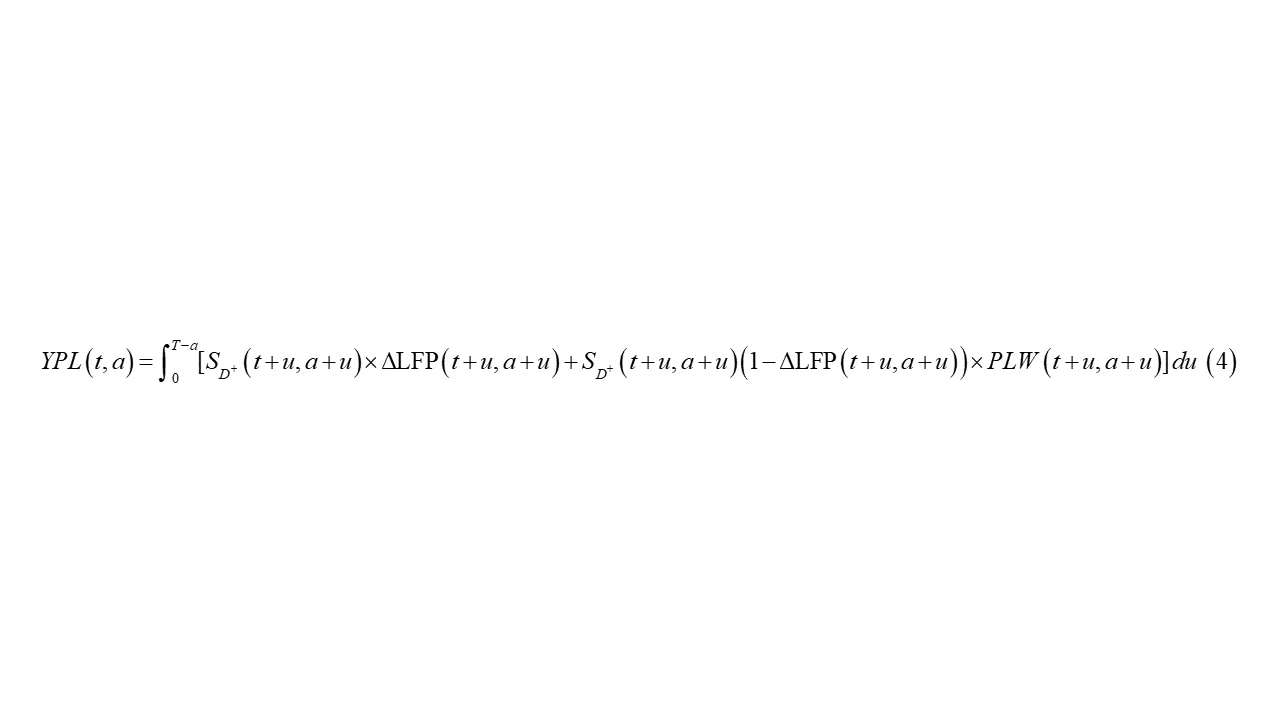



The first part of the equation represents the proportion of each year lost due to reduced labour force participation (ΔLFP), weighted by the survival probability (*S_D+_*) up to that year. The second part of the equation calculates the proportion of a year that is lost due to absenteeism and presenteeism (PLW), while accounting for the survival probability (*S_D+_*) and for the probability to participate in the labour force (1-ΔLFP), assuming that absenteeism and presenteeism are irrelevant for people outside of the labour force. Finally, PALYs loss was the sum of YLL and YPL.

We further estimated the gross domestic product (GDP) loss related to PALYs lost among individuals with type 2 diabetes. Each lost PALY was valued using China’s annual GDP per worker, sourced from the World Bank database [24]. We projected temporal trends in GDP across the model’s time horizon using the Organisation for Economic Co-operation and Development (OECD) long-term GDP forecasts [25]. For future losses accumulated over the lifetime (YLL, YPL, PALYs lost, and GDP losses), we applied the WHO standard 3% annual discount rate to the cohort-specific index year [26].

### Sensitivity and scenario analyses

To capture parameter uncertainty and *P* values for relative changes, we employed a Monte Carlo simulation with 1000 iterations. In each iteration, age- and sex-specific inputs were randomly sampled from normal distributions scaled to their respective standard errors, and the multistate illness-death model was re-run to generate projections of YLL, YPL, and PALYs lost. The resulting distribution of simulated outcomes was used to construct 95% UIs, defined by the 2.5th and 97.5th percentiles.

We also conducted sensitivity and scenario analyses to assess the impact on PALYs and GDP loss in individuals with type 2 diabetes in 2050 under different assumptions. Sensitivity analyses were undertaken by varying key parameters, including ±25% changes in productivity indices (*i.e*. absenteeism and presenteeism) and labour force dropout rates [22], alternative absenteeism estimates from Mexico or the US Centres for Disease Control and Prevention (CDC), alternative baseline prevalence from the China Chronic Disease and Risk Factors Surveillance, annual ±1% changes in incidence rates, absenteeism, presenteeism and labour force participation rate, and the upper and lower 95% confidence intervals for MRR and all-cause mortality in the general population. Scenario analyses assessed how alternative economic and policy conditions might influence the results, including:

• doubling the annual GDP growth rate

• assuming no temporal trend in GDP and maintaining 2021 GDP per worker estimates [24], and

• raising China’s retirement age to 65 for men and 60 for women [27].

In each scenario, all other model inputs were kept constant to observe the effect of modifying a single parameter on PALYs and the associated GDP losses.

All analyses and visualisations were performed using *R*, version 4.5.1 (R Foundation for Statistical Computing, Vienna, Austria). Differences between genders and between index years were tested using the Wald test. A two-sided *P* value of <0.05 was considered statistically significant.

## RESULTS

### Burden of type 2 diabetes in 2021

In 2021, 39.8 million Chinese men (9.5% of the working-age men) and 23.3 million women (6.8% of the working-age women) of working age were living with type 2 diabetes (**Table 1**; Table S1 in the **Online Supplementary Document**). Compared with individuals without diabetes, men and women experienced an excess of 216 and 79 deaths per 100,000 before retirement, respectively (Table S1 in the **Online Supplementary Document**). Over their working lifetime, type 2 diabetes reduced total years of life lived by 17.12 million in men and 3.00 million in women, equivalent to 0.43 years lost per man and 0.13 years lost per woman (Table S3 in the **Online Supplementary Document**).

**Table 1:** Mean remaining lifetime PALYs and GDP losses per prevalent working-age individual with type 2 diabetes in China, 1990–2050*

Group	1990	2000	2010	2021	2030	2040	2050
**Men**							
No. of people with T2D (in million)	12.2 (10.0–14.9)	20.1 (17.1–23.7)	28.1 (24.6–32.1)	39.8 (34.7–45.5)	48.5 (46.2–50.8)	57.9 (54.6–61.2)	56.1 (52.5–59.8)
YLL	1.11 (1.10–1.12)	0.88 (0.87–0.88)	0.63 (0.62–0.63)	0.43 (0.42–0.43)	0.37 (0.32–0.42)	0.30 (0.26–0.35)	0.24 (0.20–0.28)
YPL	0.90 (0.89–0.91)	0.96 (0.95–0.97)	0.94 (0.93–0.94)	0.91 (0.90–0.92)	0.96 (0.83–1.09)	0.97 (0.81–1.12)	0.95 (0.78–1.12)
PALY loss	2.02 (1.87–2.17)	1.84 (1.72–1.96)	1.57 (1.48–1.65)	1.34 (1.29–1.40)	1.33 (1.19–1.47)	1.27 (1.11–1.43)	1.19 (1.01–1.37)
GDP loss per person (USD)	2,660.93 (2,462.53–2,859.32)	6,786.79 (6,351.07–7,222.52)	14,595.11 (13,785.50–15,404.72)	26,038.88 (24,981.33–27,096.42)	37,552.14 (36,098.71–39,005.58)	45,466.37 (43,306.89–47,625.85)	46,905.67 (44,310.66–49,500.67)
**Women**							
No. of people with T2D (in million)	7.9 (6.4–9.7)	13.6 (11.5–16.2)	17.3 (14.9–20.0)	23.3 (20.0–27.0)	28.3 (26.7–29.9)	35.2 (32.8–37.6)	32.5 (30.1–35.0)
YLL	0.50 (0.49–0.50)	0.34 (0.33–0.34)	0.21 (0.20–0.21)	0.13 (0.13–0.13)	0.12 (0.10–0.13)	0.09 (0.08–0.11)	0.08 (0.07–0.10)
YPL	1.36 (1.34–1.37)	1.38 (1.37–1.39)	1.38 (1.37–1.39)	1.31 (1.30–1.32)	1.36 (1.16–1.56)	1.29 (1.07–1.52)	1.32 (1.06–1.58)
PALY loss	1.86 (1.69–2.02)	1.72 (1.58–1.86)	1.59 (1.47–1.72)	1.44 (1.36–1.53)	1.48 (1.27–1.68)	1.39 (1.16–1.61)	1.40 (1.14–1.66)
GDP loss per person (USD)	2,447.13 (2,226.86–2,667.41)	6,342.81 (5,812.75–6,872.87)	14,831.11 (13,695.55–15,966.67)	27,949.10 (26,310.30–29,587.89)	41,682.27 (39,455.69–43,908.86)	49,585.56 (46,426.52–52,744.60)	55,141.86 (51,162.02–59,121.70)

GDP – gross domestic product, PALYs – productivity-adjusted life years, T2D – type 2 diabetes, UI – uncertainty interval, YLL – years of life lost, YPL – years of productivity lost.

*Numbers are mean (95% UI) productive life years lost per-person with type 2 diabetes compared with a person of the same sex and age without type 2 diabetes. PALY lost are the sum of YLL and YPL. YLL, YPL, PALYs lost, and GDP losses are discounted to the cohort-specific index year at the World Health Organization standard 3% annual discount rate.

**Figure 1:**
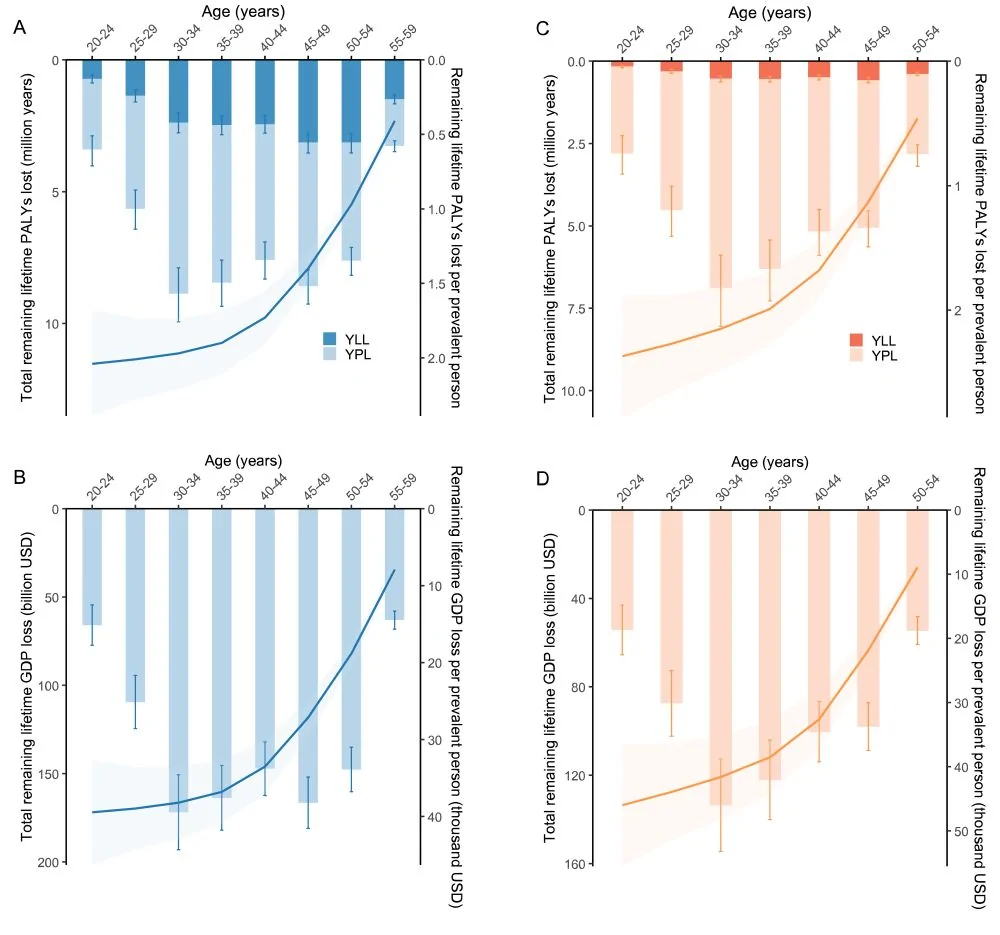
Age- and sex-specific remaining lifetime PALY and GDP losses among individuals with type 2 diabetes prevalent in 2021 in China. Panel A. Total and per-person remaining lifetime PALY loss in males. Panel B. Total and per-person remaining lifetime GDP loss in males. Panel C. Total and per-person remaining lifetime PALY loss in females. Panel D. Total and per-person remaining lifetime GDP loss in females. In all panels, bars represent total remaining lifetime losses among individuals with type 2 diabetes prevalent in each age-sex group in 2021, and lines represent corresponding per-person lifetime losses. Shaded areas indicate 95% UIs. GDP – gross domestic product, PALYs – productivity-adjusted life years, UI – uncertainty interval, YLL – years of life lost, YPL – years of productivity lost.

Type 2 diabetes was estimated to reduce lifetime PALYs among the 2021 cohort of working-age adults in China by 53.42 (95% UI = 51.25–55.59) million in men and 33.56 (95% UI = 31.59–35.52) million in women. This equated to 1.34 (95% UI = 1.29–1.40) PALYs per man and 1.44 (95% UI = 1.36–1.53) PALYs per woman, respectively (**Table 1**; Table S3 in the **Online Supplementary Document**). The total PALYs lost resulted in a GDP loss of USD 1035.31 billion in men and USD 650.38 billion in women (**Figure 1**). Across age groups, individuals aged 30–34 years had the greatest PALYs and GDP losses. Younger individuals with type 2 diabetes lost more PALYs and GDP per person than older individuals.

### Trends in productivity loss associated with type 2 diabetes from 1990 to 2021

**Figure 2:**
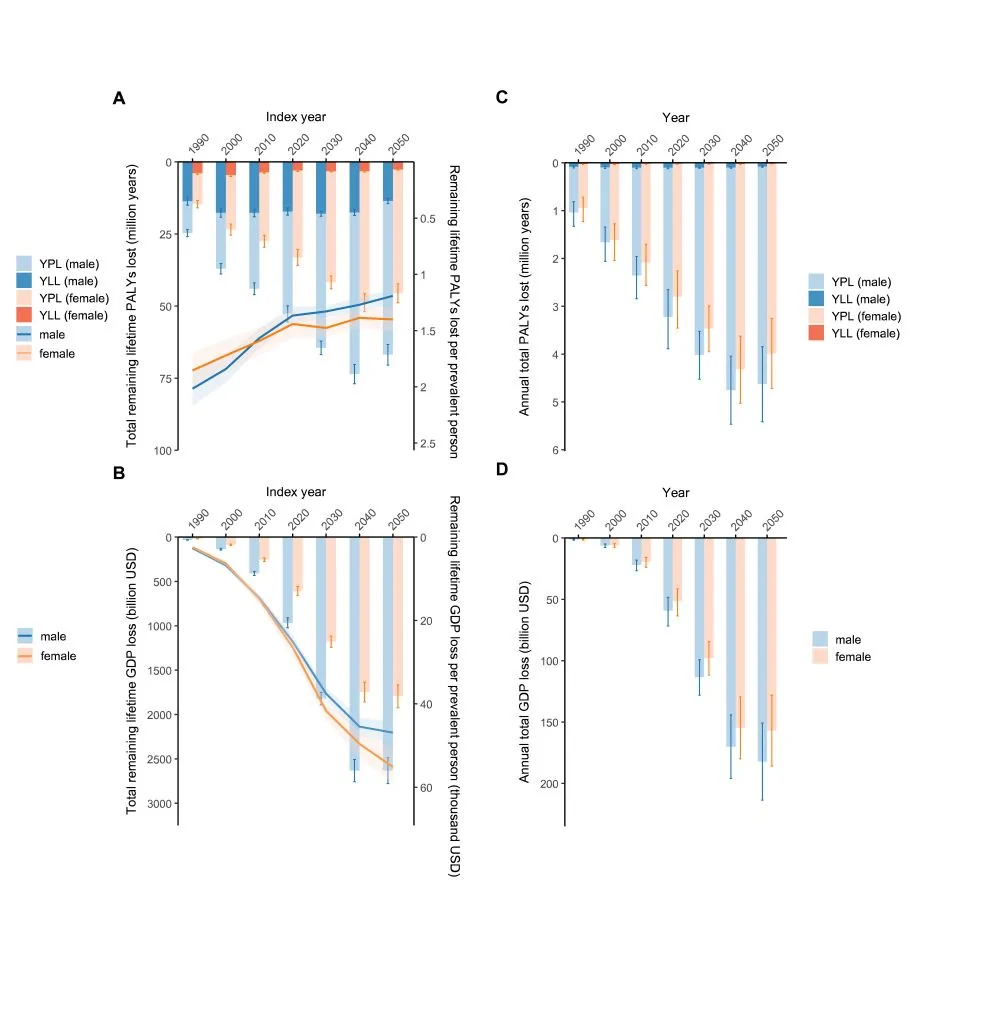
Trends in lifetime and annual cross-sectional PALYs and GDP loss in individuals with type 2 diabetes in China, 1990–2050. Panel A. Total and per-person remaining lifetime PALY loss. Panel B. Total (billion USD) and per-person (thousand USD) remaining lifetime GDP loss. Panel C. Annual cross-sectional PALYs loss. Panel D. Annual cross-sectional GDP loss. In Panels A and B, the bars represent total losses (PALYs or GDP; left y-axis), and the lines represent corresponding losses per-person (right y-axis). Shaded areas indicate 95% UIs. GDP – gross domestic product, PALYs – productivity-adjusted life years, UI – uncertainty interval, YLL – years of life lost, YPL – years of productivity lost.

For lifetime estimates, which quantify losses over the remaining working life of each cohort, productivity losses associated with type 2 diabetes increased substantially from 1990 to 2021. The number of Chinese working-age men living with type 2 diabetes more than tripled, rising from 12.2 (95% UI = 10.0–14.9) million in 1990 to 39.8 (95% UI = 34.7–45.5) million in 2021, and among women it rose from 7.9 (95% UI = 6.4–9.7) million in 1990 to 23.3 (95% UI = 20.0–27.0) million in 2021 (**Table 1**). Lifetime PALYs loss associated with type 2 diabetes increased by 116.5% (95% UI = 98.8–135.7) in males, from 24.68 million PALYs in 1990 (95% UI = 22.84–26.53) to 53.42 million PALYs in 2021 (95% UI = 51.25–55.59, *P* < 0.001). The corresponding increase in females was 128.5% (95% UI = 105.2–154.3), from 14.69 million PALYs (95% UI = 13.37–16.01) to 33.56 million PALYs (95% UI = 31.59–35.52, *P* < 0.001). By 2021, type 2 diabetes was associated with an estimated USD 1685.69 billion in losses, representing 32.5 times as much as that in the 1990 cohort of working-age adults with type 2 diabetes. At the individual level, although per-person years of life lost and PALYs lost declined, per-person GDP loss increased for both sexes. No statistically significant sex differences were observed in per-person PALYs or GDP losses over the past three decades (**Figure 2**, **Table 1**).

For population-based annual estimates, which capture losses in each calendar year, annual PALYs and GDP losses associated with type 2 diabetes also increased from 1990 to 2021 (**Figure 2**).

### Projected productivity loss associated with type 2 diabetes through 2050

**Figure 3:**
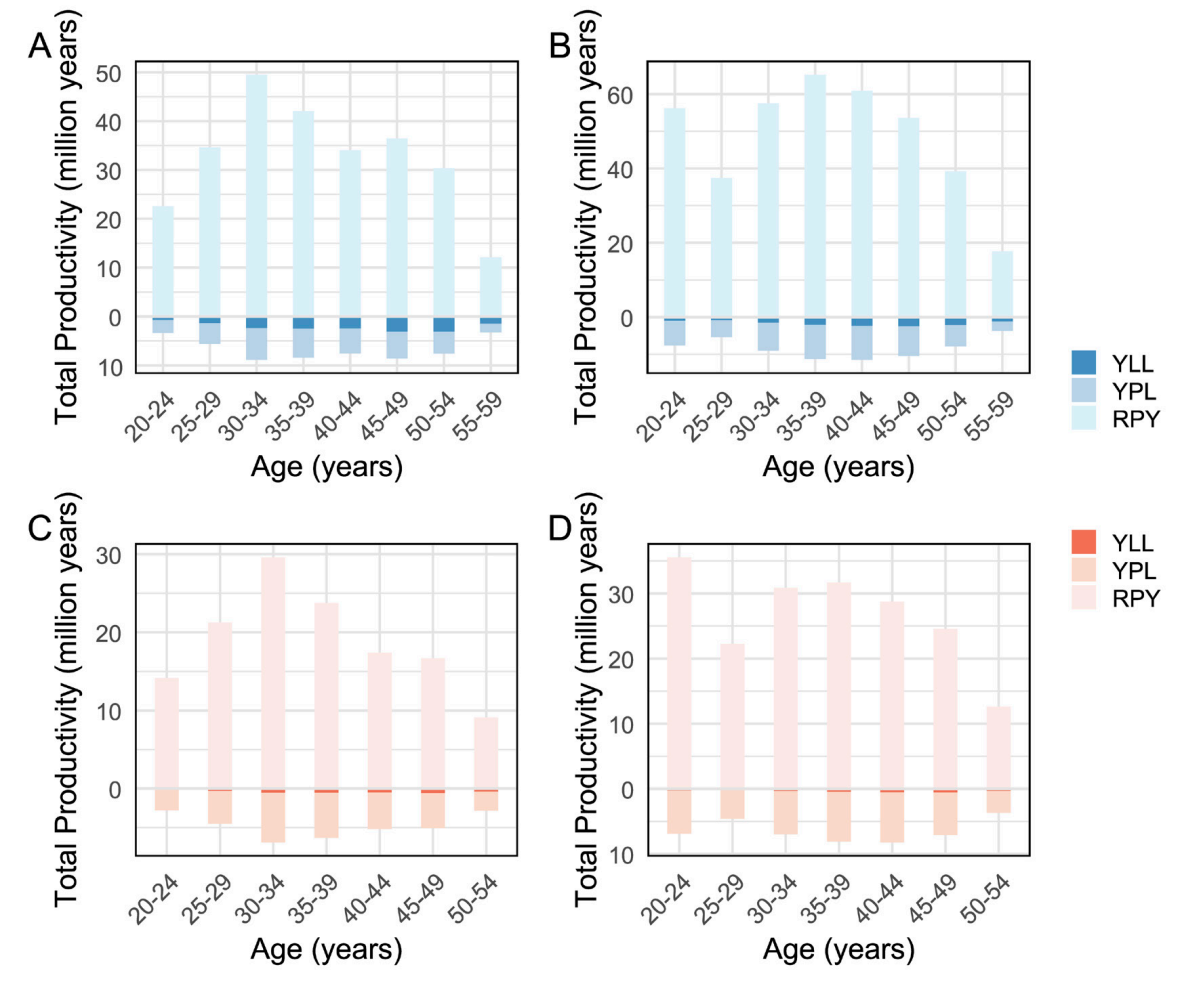
Remaining lifetime productivity years lost associated with prevalent type 2 diabetes by age and sex. Panel A. Age-specific remaining lifetime productivity years lost among working-age men with prevalent type 2 diabetes in 2021. Panel B. Age-specific remaining lifetime productivity years lost among working-age men with projected prevalent type 2 diabetes in 2050. Panel C. Age-specific remaining lifetime productivity years lost among working-age women with prevalent type 2 diabetes in 2021. Panel D. Age-specific remaining lifetime productivity years lost among working-age women with projected prevalent type 2 diabetes in 2050. YLL, YPL, and RPY (calculated as the total productive years of individuals without type 2 diabetes minus type 2 diabetes-related YLL and YPL) were all discounted at 3% annually to the corresponding index year. PALYs – productivity-adjusted life years, RPY – remaining productive years, UI – uncertainty interval, YLL – years of life lost, YPL – years of productivity lost.

For lifetime estimates, we forecast that PALYs and GDP losses associated with type 2 diabetes will increase steadily in both sexes from 2021 to 2040, followed by a slight decline from 2040 to 2050 (**Figure 2**). By 2050, 56.1 (95% UI = 52.5–59.8) million Chinese men and 32.5 (95% UI = 30.1–35.0) million women of working age are projected to live with type 2 diabetes, corresponding to an approximately 1.4-fold increase in both sexes compared with 2021 (**Table 1**). Type 2 diabetes is projected to reduce PALYs by 66.87 (95% UI = 63.17–70.57) million in men and 45.58 (95% UI = 42.29–48.87) million in women, equivalent to 1.19 (95% UI = 1.01–1.37) and 1.40 (95% UI = 1.14–1.66) PALYs loss per man and woman, respectively (**Table 1**; Table S3 in the **Online Supplementary Document**). Across age groups, premature mortality is projected to contribute more to PALYs loss in men, whereas women experience a higher proportion of PALYs loss relative to lifetime working years (**Figure 3**). At the individual level, sex differences in per-person PALYs were statistically significant in 2030 and 2050, with women exhibiting higher losses than men (**Figure 2**).

For population-based annual estimates, annual PALYs losses are projected to increase from 2021 to 2040, followed by a slight decline between 2040 and 2050. Annual GDP losses are projected to increase through 2050, with larger absolute losses in men than in women (**Figure 2**).

### Sensitivity and scenario analyses

In sensitivity and scenario analyses, a ±25% variation in labour force dropout rates altered PALYs loss in 2050 by 18.65% (Figure S3 in the **Online Supplementary Document**; Table S4 in the **Online Supplementary Document**). Assuming a 1% annual decrease in absenteeism, presenteeism, and labour force dropout rates from 2021 to 2050, total lifetime PALYs loss in the 2050 cohort could decline by 24.11 million years (Figure S4 in the **Online Supplementary Document**). Conversely, a 1% annual increase in these rates could add 31.68 million PALYs lost (Figure S5 in the **Online Supplementary Document**). Analyses using alternative absenteeism assumptions yielded broadly consistent results. Although sex differences in per-person PALYs loss were affected, overall temporal trends in total and per-person PALYs loss from 1990 to 2050 remained stable (Figure S6 in the **Online Supplementary Document**). Using alternative baseline prevalence estimates from the China Chronic Disease and Risk Factors Surveillance increased the absolute estimates of YLL, YPL, and PALYs loss from 2021 to 2050 but did not alter the general trend [28] (Table S5 in the **Online Supplementary Document**). A 1% annual increase in the incidence rates added 8.29 million PALYs lost in the 2050 cohort (Table S6 in the **Online Supplementary Document**). Doubling the annual GDP growth rate increased the projected total GDP loss in 2050 from USD 4426.68 billion to USD 8799.92 billion, whereas removing the temporal GDP trend reduced the estimated loss to USD 2179.48 billion.

Delayed retirement policy further amplifies the impact of type 2 diabetes on productivity. In the 2050 cohort, total PALYs lost increased by 42.25% relative to the baseline scenario (Table S4 in the **Online Supplementary Document**). Women experienced a larger percentage increase than men (Table S7 in the **Online Supplementary Document**). Across all age groups, PALYs lost were greater under delayed retirement, reflecting extended exposure to diabetes over a longer working life.

## DISCUSSION

Using an illness-death model, we estimated the productivity burden associated with type 2 diabetes over the working lifetime in China from 1990 to 2050. Our findings reveal a continuous increase in the total PALYs and the associated GDP losses over the past three decades, with projections suggesting a further rise through 2050. Per-person PALY and GDP losses were greater in younger than older individuals and were higher in women than men in some years, reflecting the disproportionate burden of diabetes on productivity in women and young working populations. These findings underscore the need for targeted strategies to mitigate productivity losses and address health inequities. Although retirement-age reform may expand labour supply, our findings suggest that longer working lives prolong exposure to diabetes-related productivity losses, thereby increasing lifetime PALYs and GDP losses. Retirement reform should be accompanied by policies that increase healthy working life expectancy, including effective diabetes prevention, early detection, and management, particularly among young workers and women [29–31]. Beyond macro-level reforms, sex-specific interventions are also warranted. For men, who consistently underutilise primary care and chronic disease services, improving health literacy and sustaining long-term treatment adherence may reduce premature mortality [32,33]. For women, who traditionally bear more childcare and domestic responsibilities and have higher labour force dropout rates, interventions promoting flexible work arrangements and expanding community-based childcare and elder-care services may facilitate mitigating productivity loss [22,34,35].

Expanding on previous studies [9,36,37], our research has incorporated nationally representative age- and sex-specific estimates of diabetes prevalence and incidence into a multistate illness-death model, providing a dynamic assessment and long-term projection of productivity and economic losses associated with type 2 diabetes. In our study, type 2 diabetes was associated with 53.42 million lifetime PALYs lost in men and 33.56 million in women among affected individuals in 2021, substantially exceeding previous estimates in China [9]. This discrepancy may reflect methodological differences, as the previous study assumed a retirement age of 50 years for women and did not account for incident diabetes within the cohort, which may have led to an underestimation of the productivity burden.

In China, women generally retire earlier and develop type 2 diabetes later than men [38]. Together with their lower prevalence and mortality, these factors may explain their lower years of life lost and smaller total PALYs and GDP losses compared with men [6,38,39]. Nevertheless, at the individual level, women experience higher PALYs and GDP losses per person in some years. This may be due to a higher relative risk of cardiovascular complications [40], depression, and anxiety [41], and greater caregiving responsibilities among women with diabetes [35], all of which may increase the likelihood of leaving the labour force.

Age-related disparities also warrant attention. From 1990 to 2050, diabetes-related productivity losses increase more rapidly among young adults than among older adults, with higher per-person PALYs lost in younger individuals. Early-onset type 2 diabetes may shorten life expectancy, thereby reducing the number of years that young adults would otherwise spend in the workforce [42]. Time spent in clinics and hospitals further contributes to diabetes-related absenteeism across the working lifetime. Early prevention and timely management of type 2 diabetes may confer greater productivity benefits for younger populations and help mitigate broader societal and economic burdens.

Some limitations merit consideration. First, the accuracy of the GBD estimates remains debated. Nevertheless, we used Monte Carlo simulations to generate scenario-based uncertainty intervals for both current and future estimates in China, as well as for impacts across different determinants. Second, directly measured productivity data for the Chinese population are unavailable. However, we conducted sensitivity and scenario analyses using data from several sources to vary absenteeism, presenteeism, and labour-force dropout, thereby assessing the robustness of our estimates [22]. Third, the absenteeism estimates may not be fully attributable to diabetes, although adjusted for sociodemographic characteristics, occupation, and job demands [23]. Fourth, because data on age-, sex-, and sector-specific wages and informal or unpaid work were unavailable, economic losses were valued using GDP per worker, which may not capture marginal productivity or wage heterogeneity.

## CONCLUSIONS

In conclusion, type 2 diabetes among working-age population is associated with substantial losses in PALYs, resulting in significant indirect economic costs in China. These productivity losses are projected to increase over the next three decades. Targeted interventions regarding sex- and age-related disparities are urgently needed to mitigate the growing burden.

## Additional material


Online Supplementary Document

## Data Availability

**Data availability:** All data have been obtained from publicly available sources. The analytic data set is available on request by contacting the corresponding author.
