# Supplementary material for: Productivity loss associated with type 2 diabetes in China: the trend and projection from 1990 to 2050
Source: J Glob Health. 2026 Sep 25;16:04310. doi: 10.7189/jogh.16.04310 (PMC13613105; doi:10.7189/jogh.16.04310)
Supplement: Supplementary file 1 [file jogh-16-04310-s001.pdf]

Supplement to: Zhang Y, Wu N, Zhang H, Song P, Li J, Li S. Productivity loss associated with type 2 diabetes in China: The trend and projection from 1990 to 2050. J Glob Health. 2026;16:04310.

Table of contents

Figure S1. Flow chart of the modelling framework for estimating PALYs loss associated with type 2 diabetes in China……………………………………………..………….……………....3

Figure S2. Illness-death model…………………………………………………….………….…..4

Figure S3. Sensitivity and scenario analyses of projected lifetime PALY and GDP losses among working-age individuals with type 2 diabetes in the 2050 cohort under different assumptions…….………………………………………………………………..…..…………..5

Figure S4. Sensitivity analyses of lifetime productivity years lost assuming a 1% annual decrease in absenteeism, presenteeism, and labour force dropout rates among working-age people with type 2 diabetes.………………………………………………………………….....6

Figure S5. Sensitivity analyses of lifetime productivity years lost assuming a 1% annual increase in absenteeism, presenteeism, and labour force dropout rates among working-age people with type 2 diabetes.………………………………………………………………….....7

Figure S6. Sensitivity analyses of lifetime productivity years lost using alternative sex-specific absenteeism assumptions among working-age people with type 2 diabetes…………..8

Table S1. Age- and sex-specific mortality rates in the total population, individuals with T2DM, and individuals without T2DM in China, 2021, based on Global Burden of Disease data….……………………………………………………………………..…..…….……...9

Table S2. Description and source of data used as inputs for illness-death models………...…10

Table S3. Lifetime population-level PALYs lost due to excess mortality (YLL), reduced LFP, absenteeism, and presenteeism (YPL) among people with type 2 diabetes of working age in China from 1990 to 2050…...……………………………………………………………... 11

Table S4. Sensitivity and scenario analyses to assess the impacts on PALYs and GDP loss in individuals with type 2 diabetes in working age in 2050 under different assumptions…...….13

Table S5. Comparison of sex-specific lifetime YLL, YPL, and PALYs lost between the GBD-based analysis and alternative baseline prevalence estimates from the China Chronic Disease and Risk Factors Surveillance…………………………………………...……………........14

Table S6. Lifetime population-level PALYs lost due to excess mortality (years of life lost, YLL), reduced labour force participation, absenteeism, and presenteeism (years of

productivity lost, YPL) among people with type 2 diabetes in working age in 2050 assuming 1% annual decreases and increases in the incidence rate of type 2 diabetes…..................…...15

Table S7. Lifetime population-level PALYs lost in 2050 due to excess mortality (YLL), reduced labour force participation, absenteeism, and presenteeism among people with type 2 diabetes, assuming China's delayed retirement age (65 years for men and 60 years for women) …………...…16

Checklist S1. GATHER checklist for reporting global health estimates...............................….18

Figure S1. Flow chart of the modelling framework for estimating PALYs loss associated with type 2 diabetes in China.

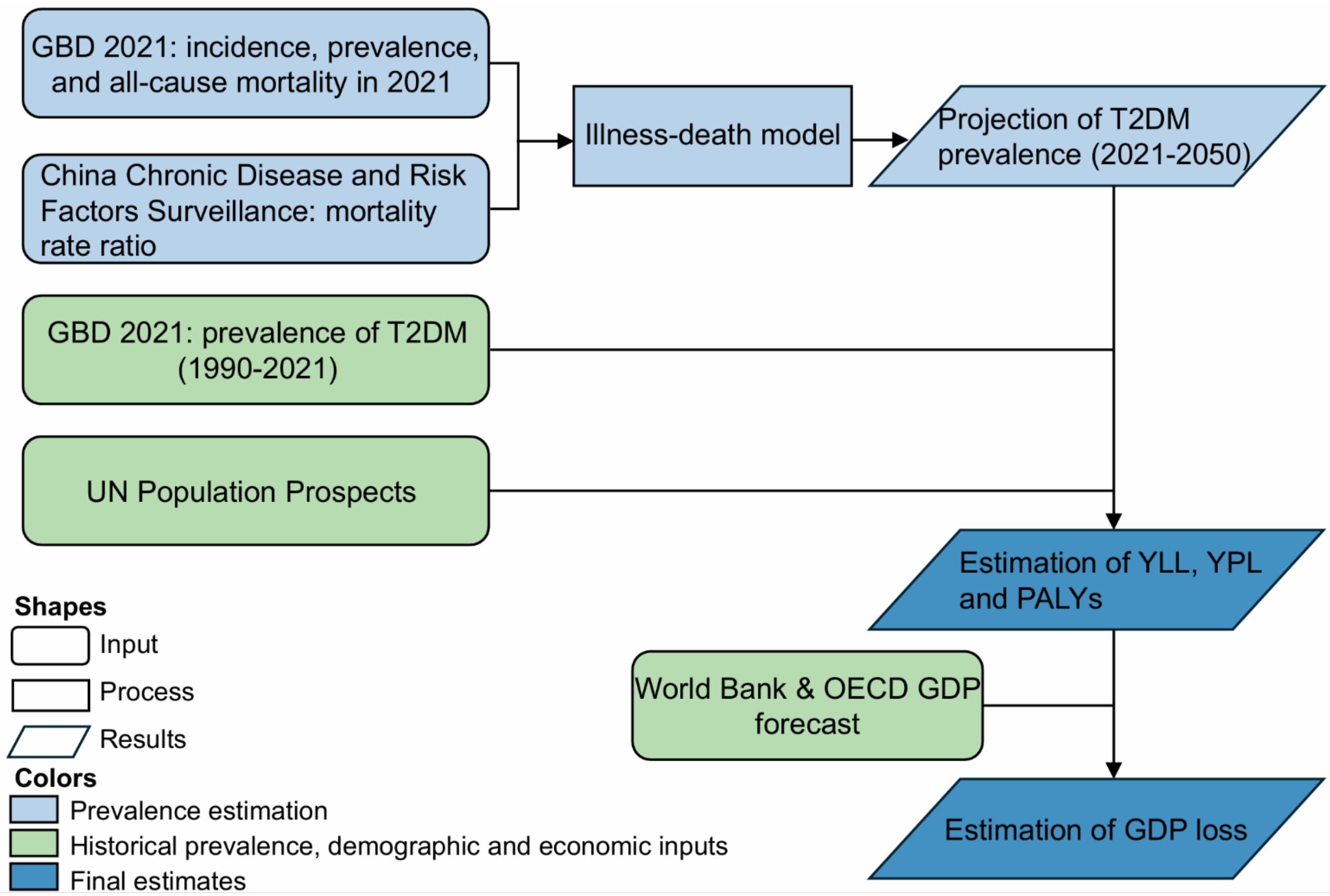

Figure S2. Illness-death model.

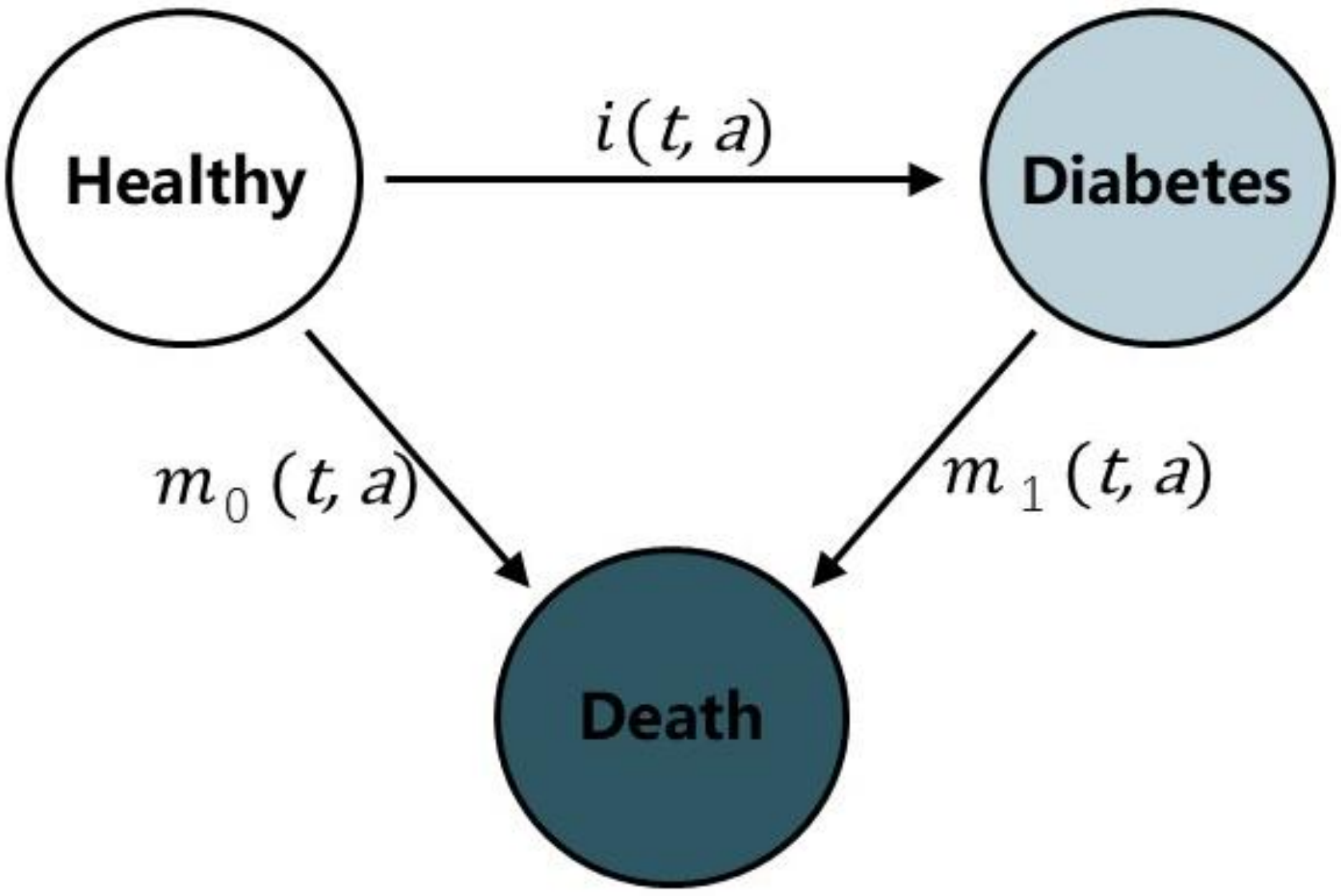

The population is divided into three states: “healthy”, “diabetes”, and “dead”. Arrows between the states indicate possible transitions: incidence rate i, mortality rate among those with type 2 diabetes m_1, mortality rate among those without type 2 diabetes m_0. In general, these rates depend on calendar time t and age a.

Figure S3. Sensitivity and scenario analyses of projected lifetime PALY and GDP losses among working-age individuals with type 2 diabetes in the 2050 cohort under different assumptions.

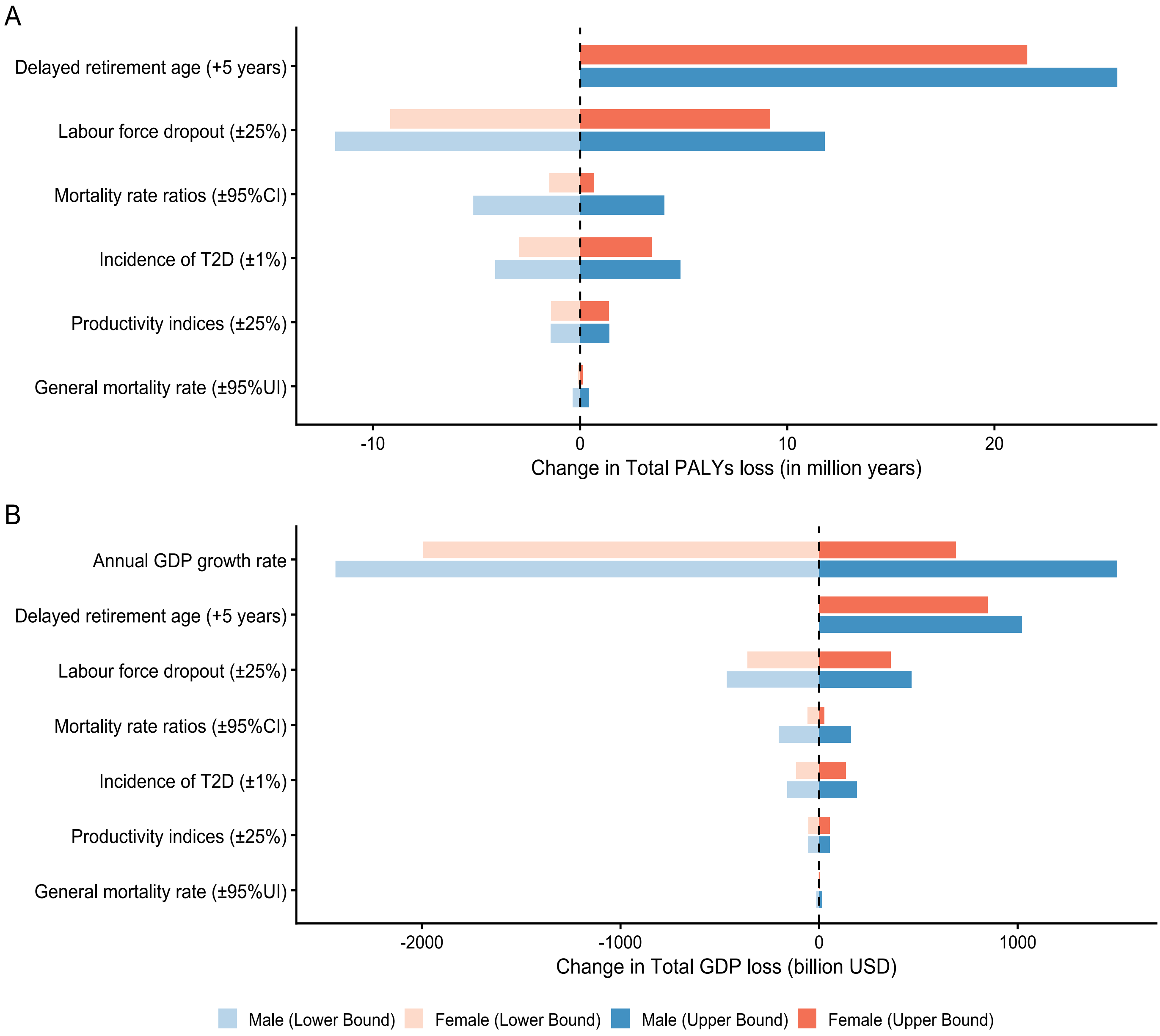

Parameters are arranged according to the widest range of model outcomes, with input value ranges shown in parentheses. Productivity indices include absenteeism and presenteeism. **Panel A.** Changes in projected lifetime total PALY loss among working-age individuals with type 2 diabetes in the 2050 cohort under different assumptions, compared with base-case estimates. **Panel B.** Changes in projected lifetime GDP loss among working-age individuals with type 2 diabetes in the 2050 cohort under different assumptions, compared with base-case estimates. For the incidence scenario, the incidence of T2D was assumed to increase or decrease by 1% annually. For the annual GDP growth-rate scenario, GDP per worker was estimated assuming either doubled annual GDP growth or no GDP growth after 2021, with GDP per worker held constant at the 2021 level. CI – confidence interval. GDP – gross domestic product. PALYs – productivity-adjusted life years. T2D – type 2 diabetes. UI – uncertainty interval.

Figure S4. Sensitivity analyses of lifetime productivity years lost assuming a 1% annual decrease in absenteeism, presenteeism, and labour force dropout rates among working-age people with type 2 diabetes.

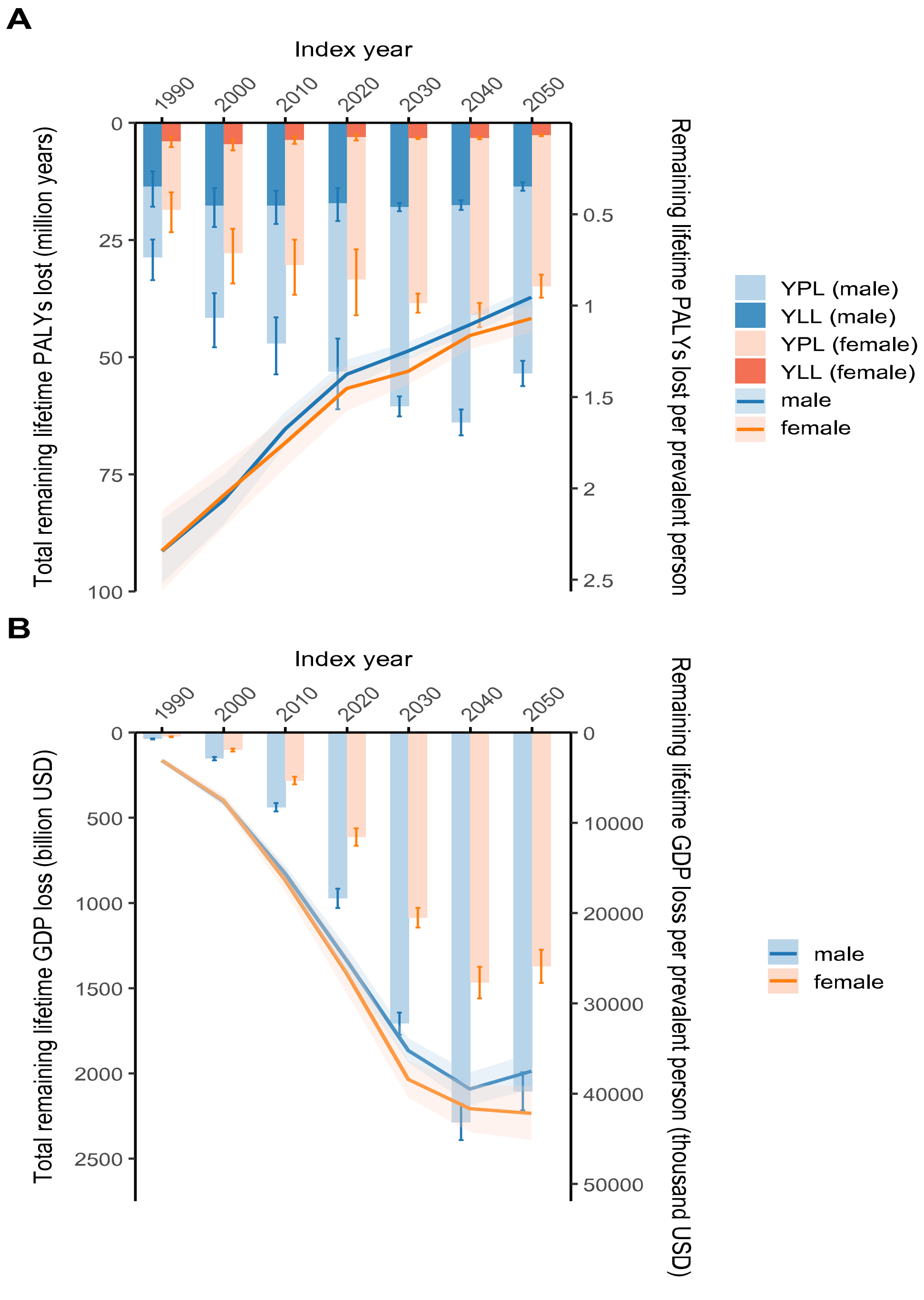

In this sensitivity analysis, these three productivity parameters were allowed to vary over time, decreasing by 1% per year across 1990-2050, with 2021 retained as the reference year. All other model parameters were held consistent with the main analysis. **Panel A.** Total PALYs loss and PALYs loss per-person; **Panel B.** Total GDP loss (billion USD) and GDP loss per-person (thousand USD). In both panels, the bars represent total losses (PALYs or GDP; left Y-axis), and the lines represent corresponding losses per-person (right Y-axis). Shaded areas indicate 95% UIs. YLL, YPL, PALYs lost, and GDP losses are discounted to the cohort-specific index year at the WHO standard 3% annual discount rate. GDP – gross domestic product. PALYs – productivity-adjusted life years. UI – uncertainty interval. YLL – years of life lost. YPL – years of productivity lost.

Figure S5. Sensitivity analyses of lifetime productivity years lost assuming a 1% annual increase in absenteeism, presenteeism, and labour force dropout rates among working-age people with type 2 diabetes.

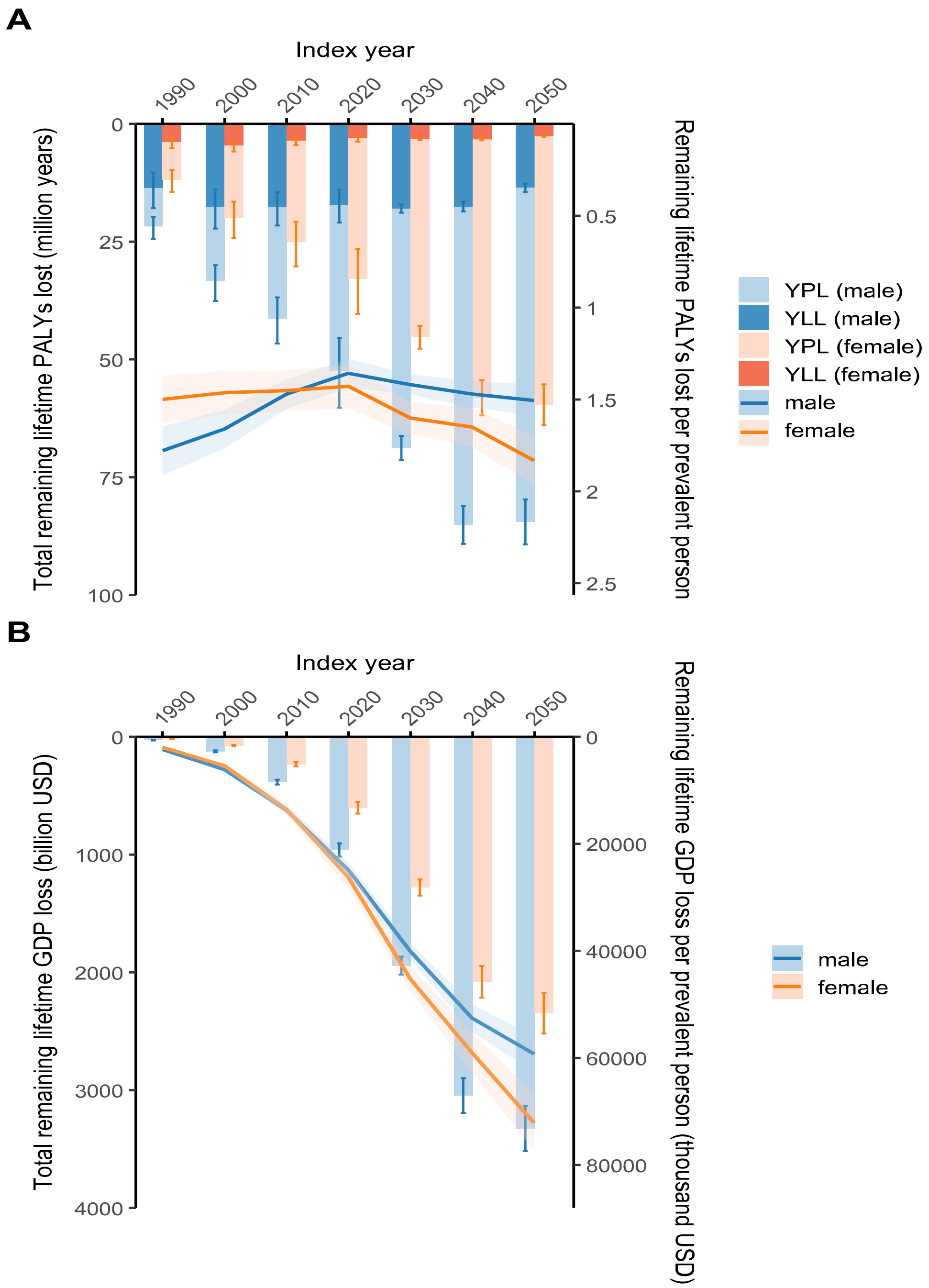

In this sensitivity analysis, these three productivity parameters were allowed to vary over time, increasing by 1% per year across 1990-2050, with 2021 retained as the reference year. All other model parameters were held consistent with the main analysis. **Panel A.** Total PALYs loss and PALYs loss per-person; **Panel B.** Total GDP loss (billion USD) and GDP loss per-person (thousand USD). In both panels, the bars represent total losses (PALYs or GDP; left Y-axis), and the lines represent corresponding losses per-person (right Y-axis). Shaded areas indicate 95% UIs. YLL, YPL, PALYs lost, and GDP losses are discounted to the cohort-specific index year at the WHO standard 3% annual discount rate. GDP – gross domestic product. PALYs – productivity-adjusted life years. UI – uncertainty interval. YLL – years of life lost. YPL – years of productivity lost.

Figure S6. Sensitivity analyses of lifetime productivity years lost using alternative sex-specific absenteeism assumptions among working-age people with type 2 diabetes.

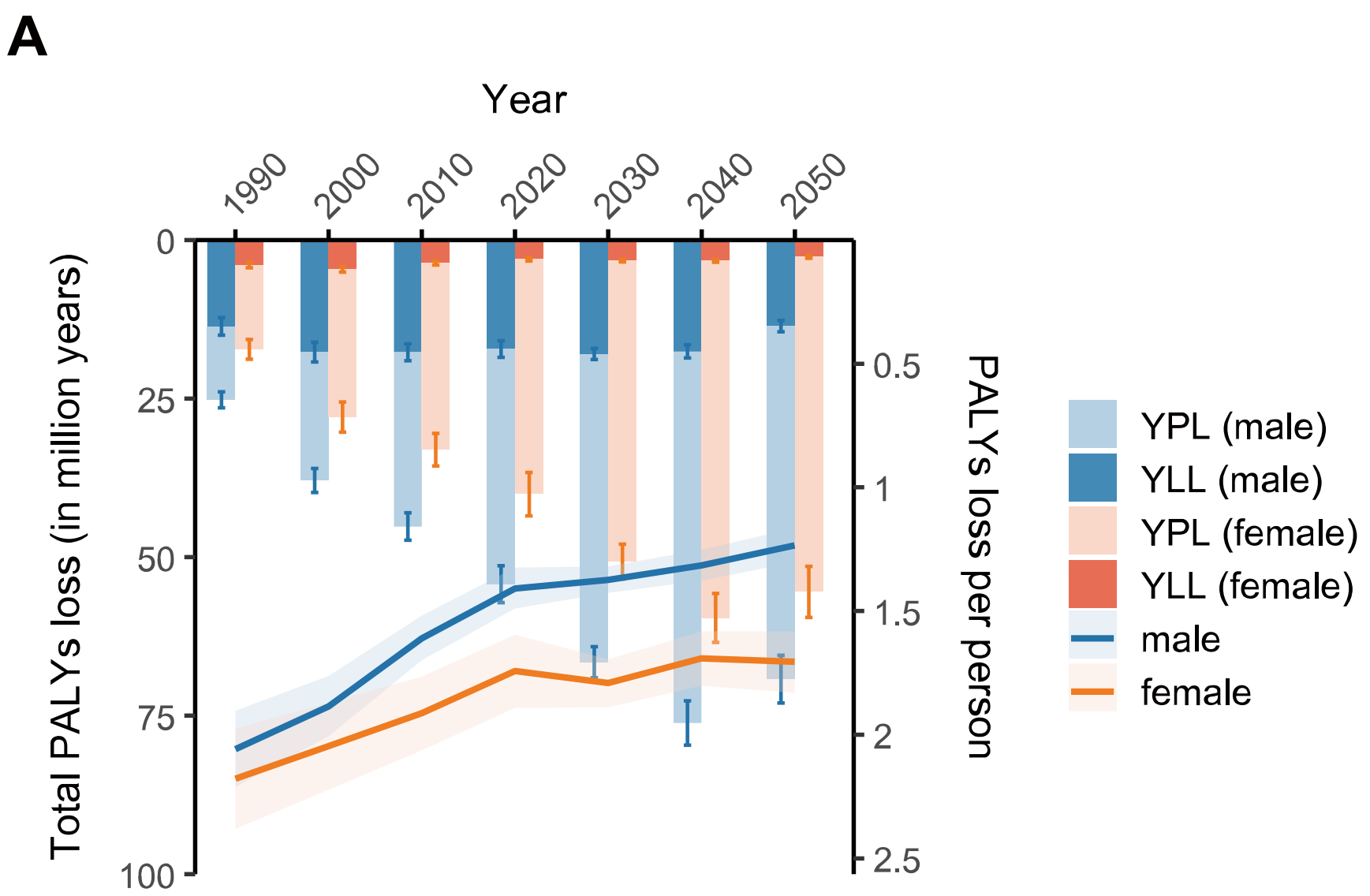

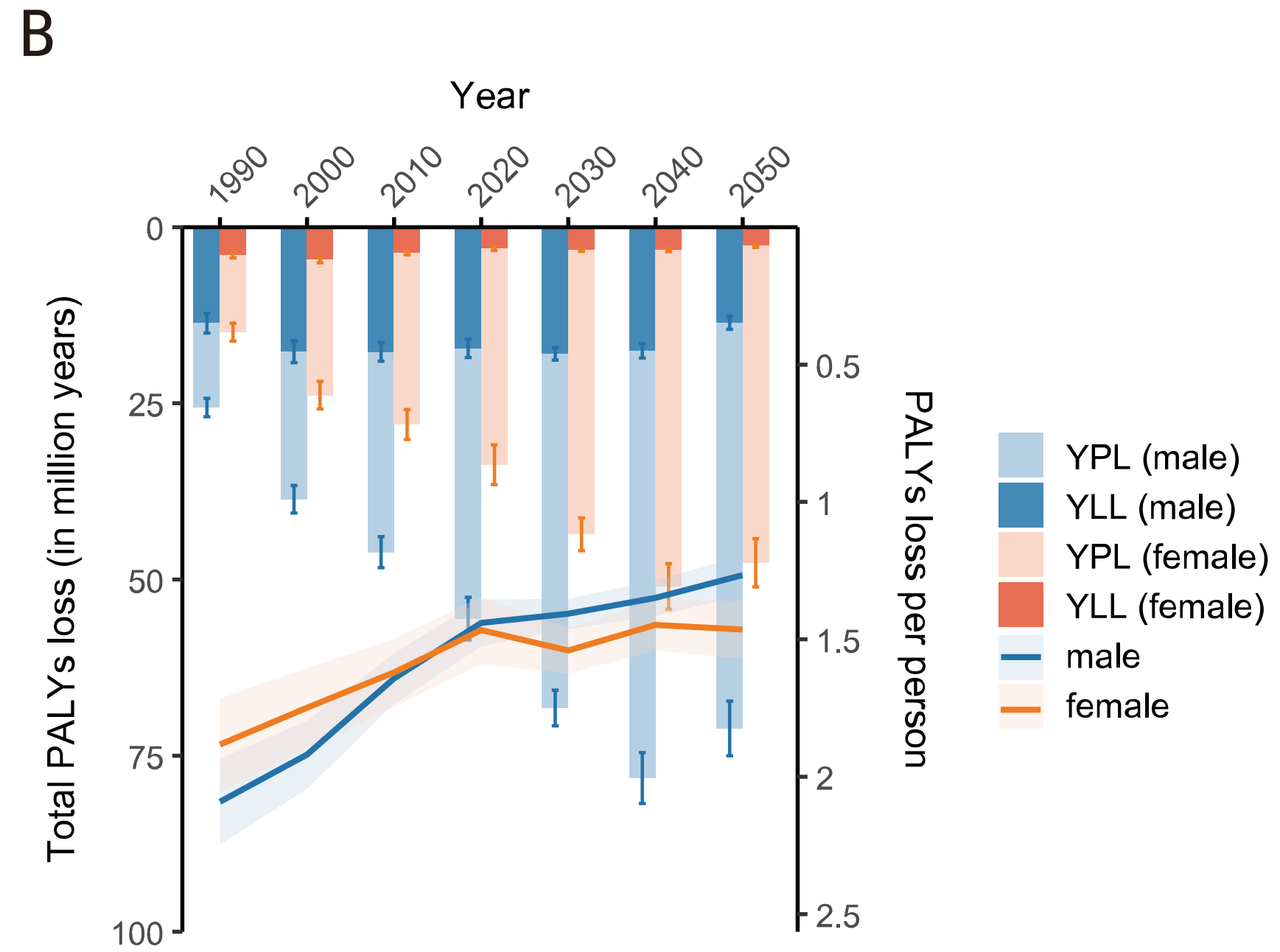

Panel A. Estimates using absenteeism parameters of 1.9 workdays per year for men and 10.2 workdays per year for women, derived from a panel data and biomarker analysis from Mexico [1,2]. **Panel B.** Estimates using absenteeism assumptions from the U.S. Centers for Disease Control and Prevention (CDC) State Burden Toolkit: 2.7 workdays lost per year for men, 2.8 workdays per year for women aged 20-44 years, and 4.7 workdays per year for women aged 45-64 years [3]. All estimates represent cumulative productivity years lost over the remaining working lifetime of individuals with prevalent type 2 diabetes in the index year. YLL, YPL, and PALYs losses are discounted to the cohort-specific index year at the WHO standard 3% annual discount rate. All other model parameters were held consistent with the main analysis.

Table S1. Age- and sex-specific mortality rates in the total population, individuals with T2DM, and individuals without T2DM in China, 2021, based on Global Burden of Disease data.

Age group	Mortality rate in the total population (per 100,000)	Prevalence of T2DM (per 100,000)	Mortality rate among individuals without T2DM (per 100,000)*	Mortality rate among individuals with T2DM (per 100,000)*
Men				
20-24	77.18	4299.99	74.83	129.46
25-29	89.82	6155.02	85.96	148.71
30-34	125.29	7184.62	119.04	205.94
35-39	177.25	8177.33	167.27	289.37
40-44	264.55	9350.87	247.65	428.42
45-49	340.91	10946.22	315.68	546.13
50-54	537.19	12842.78	491.14	849.68
55-59	800.79	14485.60	724.21	1252.88
20-59	316.04	9453.27	295.64	511.45
Women				
20-24	30.68	3438.44	29.89	52.90
25-29	32.55	4865.75	31.37	55.53
30-34	45.11	5494.25	43.28	76.60
35-39	67.11	6135.45	64.08	113.43
40-44	107.61	6894.19	102.19	180.87
45-49	149.97	8283.11	140.98	249.53
50-54	259.04	10299.28	240.01	424.81
20-54	107.29	6766.96	101.98	180.50

T2DM – type 2 diabetes mellitus.

* Mortality rates by age group and sex for individuals with and without T2DM in the Chinese population were calculated using the following formula:

$M_{total} = P*M_{dm} + (1-P)*M_{non}$

$M_{dm} = MRR*M_{non}$

Where:

M_{total} = total deaths in population/total population

M_{dm} = mortality rate in those with T2DM

M_{non} = mortality rate in those without T2DM

MRR = rate ratio of all-cause mortality in those with T2DM compared to those without T2DM

P = prevalence of T2DM

Table S2. Description and source of data used as inputs for illness-death models.

Model input and description	Data source
**Population:**	
Population of China from 1990 to 2021, stratified by sex and 5-year age groups	Global Burden of Disease (GBD) Network at https://vizhub.healthdata.org/gbd-results/.[4]
Projected population of China from 2025 to 2050, stratified by sex and 5-year age groups	United Nations, Department of Economic and Social Affairs, Population Division (2024). World Population Prospects 2024, Online Edition. [5]
**Diabetes Prevalence and Incidence:**	
Prevalence and incidence of diabetes in China in 2021, stratified by sex and 5-year age groups	Global Burden of Disease (GBD) Network at https://vizhub.healthdata.org/gbd-results/.[4]
**Mortality rates:**	
Mortality rate in the total population of China in 2021, stratified by sex and 5-year age groups	Global Burden of Disease (GBD) Network at https://vizhub.healthdata.org/gbd-results/.[4]
Rate ratio of all-cause mortality in those with T2DM compared to those without T2DM	Tian Y et al. Associations of Diabetes and Prediabetes With Mortality and Life Expectancy in China: A National Study. Diabetes Care. Nov 1 2024; 47(11):1969-1977.[6]
Temporal trends in the rate ratio of all-cause mortality in individuals with T2DM compared to those without T2DM	Carstensen B et al. Prevalence, incidence and mortality of type 1 and type 2 diabetes in Denmark 1996-2016. BMJ Open Diabetes Res Care. 2020 May;8(1):e001071.[7]
**Diabetes-related productivity losses:**	
Labour force participation shortfall, and presenteeism in those with diabetes	Bommer et al. The global economic burden of diabetes in adults aged 20-79 years: a cost-of illness study. The Lancet Diabetes & Endocrinology 2017;5:423-430.[1]
Absenteeism in those with diabetes	Tunceli K et al. The impact of diabetes on employment and work productivity. Diabetes Care. 2005 Nov;28(11):2662-7.[8]
**Gross Domestic Product (GDP):**	
The GDP and GDP per person employed in China from 1991 to 2024.	International Labour Organization, ILOSTAT database. Data retrieved in October 2025. (https://data.worldbank.org/indicator/SL.GDP. PCAP.EM.KD?locations=CN) The World Bank: World Development indicators, 2025. [9]
Temporal trends in GDP growth rate	Organisation for Economic Co-operation and Development (OECD) long-term GDP forecasts. [10]

Table S3. Lifetime population-level PALYs lost due to excess mortality (YLL), reduced LFP, absenteeism, and presenteeism (YPL) among people with type 2 diabetes of working age in China from 1990 to 2050*

Age group	1990			2021			2050		
	YLL	YPL	PALY	YLL	YPL	PALY	YLL	YPL	PALY
Men									
20-24	0.98 (0.66–1.37)	1.38 (0.94–1.94)	2.35 (1.74–2.97)	0.72 (0.58–0.88)	2.68 (2.16–3.30)	3.40 (2.81–3.99)	0.98 (0.84–1.12)	6.65 (5.69–7.55)	7.63 (6.69–8.57)
25-29	1.47 (1.07–1.99)	1.73 (1.26–2.36)	3.20 (2.48–3.92)	1.36 (1.13–1.60)	4.29 (3.58–5.06)	5.65 (4.87–6.42)	0.80 (0.61–1.00)	4.62 (3.51–5.76)	5.42 (4.28–6.56)
30-34	1.71 (1.30–2.30)	1.73 (1.31–2.32)	3.44 (2.73–4.15)	2.38 (2.02–2.77)	6.49 (5.51–7.56)	8.87 (7.77–9.96)	1.51 (1.19–1.82)	7.50 (5.92–9.03)	9.01 (7.42–10.60)
35-39	2.36 (1.78–3.07)	2.07 (1.56–2.69)	4.42 (3.56–5.28)	2.47 (2.12–2.84)	5.98 (5.13–6.88)	8.45 (7.50–9.39)	2.09 (1.67–2.48)	9.20 (7.38–10.95)	11.29 (9.46–13.12)
40-44	2.22 (1.65–2.90)	1.66 (1.24–2.17)	3.88 (3.10–4.66)	2.44 (2.11–2.78)	5.15 (4.47–5.88)	7.59 (6.81–8.38)	2.36 (2.01–2.70)	9.12 (7.75–10.44)	11.48 (10.09–12.88)
45-49	2.05 (1.62–2.58)	1.23 (0.97–1.55)	3.28 (2.72–3.84)	3.13 (2.78–3.53)	5.46 (4.84–6.14)	8.59 (7.84–9.34)	2.49 (2.04–2.99)	7.93 (6.50–9.53)	10.42 (8.83–12.01)
50-54	1.90 (1.51–2.45)	0.90 (0.72–1.16)	2.80 (2.28–3.32)	3.13 (2.79–3.53)	4.49 (3.99–5.05)	7.62 (6.97–8.27)	2.18 (1.82–2.52)	5.73 (4.80–6.64)	7.91 (6.92–8.89)
55-59	0.95 (0.75–1.22)	0.36 (0.29–0.47)	1.31 (1.06–1.56)	1.49 (1.33–1.67)	1.77 (1.58–1.99)	3.25 (2.99–3.52)	1.16 (0.97–1.35)	2.54 (2.13–2.95)	3.70 (3.26–4.15)
20-59	13.63 (12.25–15.01)	11.06 (9.84–12.28)	24.68 (22.84–26.53)	17.12 (16.24–17.99)	36.30 (34.32–38.29)	53.42 (51.25–55.59)	13.57 (12.66–14.48)	53.30 (49.71–56.89)	66.87 (63.17–70.57)

Table S3. (continued)

Age group	1990			2021			2050		
	YLL	YPL	PALY	YLL	YPL	PALY	YLL	YPL	PALY
Women									
20-24	0.39 (0.27–0.54)	1.63 (1.13–2.25)	2.03 (1.45–2.60)	0.16 (0.13–0.20)	2.64 (2.10–3.27)	2.80 (2.22–3.38)	0.26 (0.22–0.30)	6.63 (5.54–7.67)	6.89 (5.83–7.96)
25-29	0.55 (0.40–0.74)	1.91 (1.41–2.58)	2.46 (1.85–3.07)	0.31 (0.25–0.37)	4.21 (3.48–5.01)	4.51 (3.75–5.28)	0.20 (0.15–0.26)	4.40 (3.31–5.57)	4.61 (3.47–5.74)
30-34	0.59 (0.45–0.78)	1.77 (1.35–2.37)	2.36 (1.82–2.90)	0.53 (0.45–0.63)	6.36 (5.36–7.52)	6.89 (5.81–7.97)	0.35 (0.27–0.43)	6.62 (5.11–8.05)	6.96 (5.49–8.44)
35-39	0.78 (0.59–1.02)	2.11 (1.59–2.76)	2.89 (2.26–3.51)	0.54 (0.46–0.63)	5.77 (4.89–6.74)	6.30 (5.37–7.23)	0.45 (0.36–0.54)	7.66 (6.06–9.20)	8.11 (6.54–9.68)
40-44	0.67 (0.51–0.88)	1.61 (1.21–2.11)	2.29 (1.80–2.78)	0.49 (0.42–0.57)	4.68 (4.01–5.41)	5.18 (4.47–5.88)	0.51 (0.43–0.59)	7.71 (6.45–8.89)	8.22 (7.00–9.45)
45-49	0.58 (0.46–0.74)	1.13 (0.89–1.43)	1.72 (1.41–2.02)	0.58 (0.51–0.66)	4.48 (3.96–5.06)	5.06 (4.50–5.61)	0.54 (0.43–0.66)	6.56 (5.22–8.04)	7.09 (5.68–8.51)
50-54	0.37 (0.28–0.49)	0.58 (0.45–0.76)	0.94 (0.76–1.13)	0.39 (0.34–0.44)	2.43 (2.15–2.80)	2.82 (2.49–3.14)	0.33 (0.28–0.39)	3.35 (2.83–3.86)	3.69 (3.17–4.21)
20-54	3.93 (3.50–4.37)	10.76 (9.51–12.01)	14.69 (13.37–16.01)	3.00 (2.82–3.18)	30.56 (28.60–32.52)	33.56 (31.59–35.52)	2.64 (2.44–2.85)	42.94 (39.65–46.22)	45.58 (42.29–48.87)

PALYs – productivity-adjusted life years. YLL – years of life lost. YPL – years of productivity lost.

*Numbers are in million years (95%UIs). PALY lost are the sum of YLL and YPL. YLL, YPL, and PALYs lost are discounted to the cohort-specific index year at the WHO standard 3% annual discount rate.

Table S4. Sensitivity and scenario analyses to assess the impacts on PALYs and GDP loss in individuals with type 2 diabetes in working age in 2050 under different assumptions.

Scenario	PALYs loss due to T2DM (million years)	% change in PALYs lost compared with base case	GDP loss (billion USD)	GDP lost per person with T2DM (USD)
Base case	112.45	0	4426.68	49928
(1) Productivity indices upper uncertainty bound *	115.24	2.48	4536.51	51167
(2) Productivity indices lower uncertainty bound *	109.65	-2.48	4316.45	48685
(3) Labour force dropout upper uncertainty bound †	133.42	18.65	5252.18	59239
(4) Labour force dropout lower uncertainty bound †	91.47	-18.65	3600.78	40613
(5) Mortality rate ratios upper uncertainty bound ‡	117.19	4.22	4613.27	52033
(6) Mortality rate ratios lower uncertainty bound ‡	105.80	-5.91	4164.90	46976
(7) Incidence of T2DM upper uncertainty bound §	120.74	7.37	4753.02	53609
(8) Incidence of T2DM lower uncertainty bound §	105.42	-6.25	4149.94	46807
(9) General mortality rate upper uncertainty bound ¶	111.89	-0.50	4404.63	49680
(10) General mortality rate lower uncertainty bound ¶	112.91	0.41	4444.79	50132
(11) No temporal trend in GDP	112.45	0	2179.48	24582
(12) Doubled annual GDP growth rate	112.45	0	8799.92	99254
(13) Delayed retirement age ‖	159.96	42.25	6296.94	71023

* Applied (1) a 25% increase and (2) a 25% decrease in absenteeism and presenteeism estimates, holding all other model inputs constant. †Applied (3) a 25% increase and (4) a 25% decrease in labour force dropout estimates, holding all other model inputs constant. ‡Applied (5) the upper bound of the 95%CI and (6) the lower bound of the 95%CI around the estimate of MRR, holding all other model inputs constant. §Applied (7) a 1% annual increase and (8) a 1% annual decrease in incidence of T2DM, holding all other model inputs constant. ¶Applied (9) the upper bound of the 95%UI and (10) the lower bound of the 95%UI around the all-cause mortality in the general population, holding all other model inputs constant. ‖Applied China's delayed retirement age (65 years for men and 60 years for women). PALYs and GDP losses are discounted to the cohort-specific index year at the WHO standard 3% annual discount rate.

Table S5. Comparison of sex-specific lifetime YLL, YPL, and PALYs lost between the GBD-based analysis and alternative baseline prevalence estimates from the China Chronic Disease and Risk Factors Surveillance*

Index year	male			female		
	YLL	YPL	PALY	YLL	YPL	PALY
GBD						
2021	17.12 (16.24–17.99)	36.30 (34.32–38.29)	53.42 (51.25–55.59)	3.00 (2.82–3.18)	30.56 (28.60–32.52)	33.56 (31.59–35.52)
2030	17.98 (17.10–18.86)	46.51 (44.18–48.85)	64.49 (62.00–66.99)	3.26 (3.08–3.45)	38.50 (36.28–40.73)	41.76 (39.53–44.00)
2040	17.56 (16.53–18.59)	56.04 (52.70–59.38)	73.60 (70.10–77.09)	3.27 (3.05–3.49)	45.57 (42.46–48.67)	48.84 (45.73–51.95)
2050	13.57 (12.66–14.48)	53.30 (49.71–56.89)	66.87 (63.17–70.57)	2.64 (2.44–2.85)	42.94 (39.65–46.22)	45.58 (42.29–48.87)
2018-2019 China Chronic Disease and Risk Factors Surveillance [11]						
2021	24.08 (22.77–25.42)	51.08 (48.10–54.11)	75.16 (70.90–79.49)	5.10 (4.77–5.43)	51.94 (48.40–55.56)	57.04 (53.17–60.97)
2030	23.38 (22.20–24.55)	60.11 (56.94–63.29)	83.49 (79.15–87.81)	4.91 (4.61–5.20)	57.65 (54.12–61.19)	62.56 (58.73–66.39)
2040	21.60 (20.46–22.71)	68.40 (64.70–72.08)	90.00 (85.19–94.75)	4.50 (4.24–4.76)	62.37 (58.60–66.20)	66.87 (62.84–70.95)
2050	16.19 (15.23–17.14)	63.27 (59.51–67.05)	79.46 (74.76–84.19)	3.48 (3.26–3.70)	56.37 (52.71–60.07)	59.85 (55.98–63.76)

PALYs – productivity-adjusted life years. YLL – years of life lost. YPL – years of productivity lost.

*Estimates represent cumulative losses over the remaining working lifetime of individuals prevalent in each index year. Numbers are in million years (95%UIs). PALY lost are the sum of YLL and YPL. YLL, YPL, and PALYs lost are discounted to the cohort-specific index year at the WHO standard 3% annual discount rate.

Table S6. Lifetime population-level PALYs lost due to excess mortality (years of life lost, YLL), reduced labour force participation, absenteeism, and presenteeism (years of productivity lost, YPL) among people with type 2 diabetes in working age in 2050 assuming 1% annual decreases and increases in the incidence rate of type 2 diabetes*

Age group	Decreasing incidence scenario			Increasing incidence scenario		
	YLL	YPL	PALY	YLL	YPL	PALY
Men						
20-24	0.89 (0.76–1.01)	6.03 (5.16–6.86)	6.92 (6.07–7.78)	1.09 (0.93–1.24)	7.37 (6.32–8.38)	8.46 (7.42–9.50)
25-29	0.75 (0.58–0.93)	4.32 (3.36–5.34)	5.08 (4.07–6.08)	0.86 (0.64–1.09)	4.96 (3.69–6.25)	5.83 (4.53–7.12)
30-34	1.43 (1.13–1.70)	7.10 (5.63–8.47)	8.53 (7.08–9.98)	1.60 (1.26–1.95)	7.97 (6.26–9.71)	9.58 (7.82–11.34)
35-39	1.97 (1.60–2.33)	8.70 (7.05–10.27)	10.67 (9.03–12.32)	2.22 (1.76–2.66)	9.79 (7.77–11.74)	12.02 (9.98–14.05)
40-44	2.23 (1.90–2.53)	8.59 (7.34–9.78)	10.82 (9.56–12.08)	2.53 (2.12–2.90)	9.75 (8.20–11.18)	12.28 (10.74–13.82)
45-49	2.34 (1.94–2.78)	7.45 (6.18–8.88)	9.79 (8.37–11.20)	2.67 (2.15–3.23)	8.51 (6.87–10.31)	11.17 (9.37–12.97)
50-54	2.05 (1.73–2.37)	5.40 (4.56–6.23)	7.45 (6.56–8.34)	2.33 (1.93–2.71)	6.12 (5.07–7.11)	8.44 (7.35–9.53)
55-59	1.10 (0.93–1.27)	2.41 (2.04–2.78)	3.51 (3.11–3.92)	1.23 (1.02–1.43)	2.70 (2.24–3.14)	3.93 (3.43–4.42)
20-59	12.76 (11.94–13.58)	50.02 (46.78–53.25)	62.77 (59.44–66.11)	14.52 (13.51–15.54)	57.17 (53.17–61.18)	71.70 (67.57–75.83)
Women						
20-24	0.23 (0.20–0.27)	6.01 (5.02–6.95)	6.24 (5.28–7.21)	0.29 (0.24–0.33)	7.36 (6.14–8.53)	7.65 (6.46–8.84)
25-29	0.19 (0.15–0.24)	4.13 (3.17–5.15)	4.32 (3.33–5.31)	0.22 (0.16–0.28)	4.73 (3.45–6.02)	4.95 (3.66–6.23)
30-34	0.33 (0.26–0.40)	6.28 (4.90–7.58)	6.61 (5.27–7.95)	0.37 (0.28–0.45)	7.02 (5.38–8.61)	7.39 (5.77–9.01)
35-39	0.43 (0.34–0.51)	7.27 (5.78–8.67)	7.69 (6.25–9.14)	0.48 (0.37–0.58)	8.13 (6.32–9.86)	8.61 (6.84–10.38)
40-44	0.48 (0.41–0.55)	7.25 (6.12–8.32)	7.73 (6.63–8.84)	0.55 (0.46–0.64)	8.26 (6.89–9.56)	8.81 (7.47–10.15)
45-49	0.50 (0.40–0.61)	6.11 (4.93–7.43)	6.61 (5.36–7.86)	0.58 (0.45–0.71)	7.08 (5.55–8.75)	7.66 (6.05–9.26)
50-54	0.31 (0.27–0.36)	3.13 (2.66–3.59)	3.44 (2.97–3.91)	0.36 (0.30–0.42)	3.62 (3.04–4.17)	3.98 (3.41–4.54)
20-54	2.48 (2.29–2.66)	40.17 (37.21–43.14)	42.65 (39.68–45.62)	2.84 (2.61–3.07)	46.20 (42.53–49.87)	49.04 (45.36–52.72)

PALYs – productivity-adjusted life years. YLL – years of life lost. YPL – years of productivity lost.

*Numbers are in million years (95%UIs). PALY lost are the sum of YLL and YPL. YLL, YPL, and PALYs lost are discounted to the cohort-specific index year at the WHO standard 3% annual discount rate.

Table S7. Lifetime population-level PALYs lost in 2050 due to excess mortality (YLL), reduced labour force participation, absenteeism, and presenteeism among people with type 2 diabetes, assuming China's delayed retirement age (65 years for men and 60 years for women) *

Age group	2050		
	YLL	YPL	PALY
Men			
20-24	1.29 (1.11–1.47)	7.12 (6.10–8.09)	8.42 (7.40–9.43)
25-29	1.07 (0.81–1.33)	5.01 (3.81–6.26)	6.08 (4.83–7.33)
30-34	2.03 (1.60–2.44)	8.30 (6.54–9.98)	10.33 (8.55–12.10)
35-39	2.88 (2.31–3.43)	10.41 (8.35–12.39)	13.29 (11.20–15.38)
40-44	3.40 (2.89–3.90)	10.71 (9.10–12.26)	14.11 (12.45–15.77)
45-49	3.87 (3.17–4.65)	10.04 (8.22–12.06)	13.91 (11.85–15.96)
50-54	3.97 (3.33–4.60)	8.45 (7.08–9.79)	12.42 (10.92–13.92)
55-59	3.52 (2.95–4.08)	6.13 (5.13–7.10)	9.65 (8.51–10.78)
60-64	1.89 (1.61–2.17)	2.71 (2.31–3.12)	4.60 (4.11–5.10)
20-64	23.92 (22.44–25.41)	68.87 (64.55–73.19)	92.80 (88.23–97.36)
Women			
20-24	0.37 (0.31–0.42)	7.34 (6.14–8.50)	7.71 (6.53–8.89)
25-29	0.29 (0.22–0.37)	4.98 (3.74–6.30)	5.27 (3.99–6.55)
30-34	0.51 (0.39–0.62)	7.69 (5.94–9.36)	8.20 (6.49–9.92)
35-39	0.68 (0.54–0.82)	9.23 (7.31–11.09)	9.92 (8.02–11.81)
40-44	0.84 (0.70–0.97)	9.90 (8.28–11.41)	10.74 (9.17–12.31)
45-49	1.03 (0.82–1.26)	9.85 (7.84–12.08)	10.88 (8.75–13.00)
50-54	1.07 (0.90–1.23)	8.27 (6.97–9.51)	9.33 (8.05–10.62)
55-59	0.70 (0.59–0.80)	4.41 (3.72–5.09)	5.11 (4.42–5.80)
20-59	5.47 (5.09–5.86)	61.68 (57.38–65.99)	67.16 (62.84–71.48)

PALYs – productivity-adjusted life years. YLL – years of life lost. YPL – years of productivity lost.

*Numbers are in million years (95%UIs). PALY lost are the sum of YLL and YPL. YLL, YPL, and PALYs lost are discounted to the cohort-specific index year at the WHO standard 3% annual discount rate.

Electronic Supplementary Material references:

1. Bommer C, Heesemann E, Sagalova V, Manne-Goehler J, Atun R, Bärnighausen T, et al. The global economic burden of diabetes in adults aged 20-79 years: a cost-of-illness study. Lancet Diabetes Endocrinol. 2017;5:423-30.
2. Seuring T, Serneels P, Suhrcke M. The impact of diabetes on labor market outcomes in Mexico: A panel data and biomarker analysis. IZA Discussion Paper No. 10123. Bonn, Germany: Institute for the Study of Labor; 2016.
3. Centers for Disease Control and Prevention. State Burden Toolkit: Economic burden. 18 September 2025. Available: https://www.cdc.gov/diabetes/burden-toolkit/state-burden-toolkit-economic-burden.html?cove-tab=2. Accessed: 5 May 2026.
4. Global Burden of Disease Collaborative Network. Global Burden of Disease Study 2021 (GBD 2021) Results [dataset]. 2024. Available: https://vizhub.healthdata.org/gbd-results/. Accessed: 4 July 2025.
5. United Nations, Department of Economic and Social Affairs, Population Division. World Population Prospects 2024, Online Edition. Available: https://population.un.org/wpp/. Accessed: 10 July 2025.
6. Tian Y, Qiu Z, Wang F, Deng S, Wang Y, Wang Z, et al. Associations of diabetes and prediabetes with mortality and life expectancy in China: A national study. Diabetes Care. 2024;47:1969-77.
7. Carstensen B, Rønn PF, Jørgensen ME. Prevalence, incidence and mortality of type 1 and type 2 diabetes in Denmark 1996-2016. BMJ Open Diabetes Res Care. 2020;8:e001071.
8. Tunceli K, Bradley CJ, Nerenz D, Williams LK, Pladevall M, Lafata JE. The impact of diabetes on employment and work productivity. Diabetes Care. 2005;28:2662-7.
9. The World Bank. World Development Indicators. 2025. Available: https://databank.worldbank.org/source/world-development-indicators. Accessed: 8 October 2025.
10. Organisation for Economic Co-operation and Development. OECD Economic Outlook: Statistics and Projections: Long-term baseline projections, Real GDP long-term forecast (indicator). 2025. Available: https://www.oecd.org/en/data/indicators/real-gdp-long-term-forecast.html. Accessed: 8 October 2025.
11. Wang L, Peng W, Zhao Z, Zhang M, Shi Z, Song Z, et al. Prevalence and Treatment of Diabetes in China, 2013-2018. JAMA. 2021;326:2498-506.

Checklist S1. GATHER checklist for reporting global health estimates.

Checklist of information that should be included in new reports of global health estimates

Item #	Checklist item	Reported on page #
**Objectives and funding**		
**1**	Define the indicator(s), populations (including age, sex, and geographic entities), and time period(s) for which estimates were made.	Page3-4
**2**	List the funding sources for the work.	Page14
**Data Inputs**		
For all data inputs from multiple sources that are synthesized as part of the study:		
**3**	Describe how the data were identified and how the data were accessed.	Page3-4
**4**	Specify the inclusion and exclusion criteria. Identify all ad-hoc exclusions.	Page3-4
**5**	Provide information on all included data sources and their main characteristics. For each data source used, report reference information or contact name/institution, population represented, data collection method, year(s) of data collection, sex and age range, diagnostic criteria or measurement method, and sample size, as relevant.	Page3-4; Table S2
**6**	Identify and describe any categories of input data that have potentially important biases (e.g., based on characteristics listed in item 5).	Page13
For data inputs that contribute to the analysis but were not synthesized as part of the study:		
**7**	Describe and give sources for any other data inputs.	Page7
For all data inputs:		
**8**	Provide all data inputs in a file format from which data can be efficiently extracted (e.g., a spreadsheet rather than a PDF), including all relevant meta-data listed in item 5. For any data inputs that cannot be shared because of ethical or legal reasons, such as third-party ownership, provide a contact name or the name of the institution that retains the right to the data.	Page13
**Data analysis**		
**9**	Provide a conceptual overview of the data analysis method. A diagram may be helpful.	Page3;FigureS1
**10**	Provide a detailed description of all steps of the analysis, including mathematical formulae. This description should cover, as relevant, data cleaning, data pre-processing, data adjustments and weighting of data sources, and mathematical or statistical model(s).	Page3-8
**11**	Describe how candidate models were evaluated and how the final model(s) were selected.	Page3;Page5
**12**	Provide the results of an evaluation of model performance, if done, as well as the results of any relevant sensitivity analysis.	Page10-11
**13**	Describe methods for calculating uncertainty of the estimates. State which sources of uncertainty were, and were not, accounted for in the uncertainty analysis.	Page7
**14**	State how analytic or statistical source code used to generate estimates can be accessed.	Page13
**Results and Discussion**		
**15**	Provide published estimates in a file format from which data can be efficiently extracted.	Table1
**16**	Report a quantitative measure of the uncertainty of the estimates (e.g. uncertainty intervals).	Page8-10
**17**	Interpret results in light of existing evidence. If updating a previous set of estimates, describe the reasons for changes in estimates.	Page12
**18**	Discuss limitations of the estimates. Include a discussion of any modelling assumptions or data limitations that affect interpretation of the estimates.	Page13

This checklist should be used in conjunction with the GATHER statement and Explanation and Elaboration document, found on gather-statement.org
